# Vertical RAS pathway inhibition in pancreatic cancer drives therapeutically exploitable mitochondrial alterations

**DOI:** 10.1038/s41392-025-02563-7

**Published:** 2026-01-16

**Authors:** Philipp Hafner, Steffen J. Keller, Xun Chen, Asma Alrawashdeh, Huda Jumaa, Friederike I. Nollmann, Solène Besson, Judith Kemming, Oliver Gorka, Tonmoy Das, Bismark Appiah, Ariane Lehmann, Mujia Li, Petya Apostolova, Bertram Bengsch, Robert Zeiser, Stefan Tholen, Oliver Schilling, Olaf Groß, Andreas Vlachos, Uwe A. Wittel, Dominik von Elverfeldt, Wilfried Reichardt, Melanie Boerries, Geoffroy Andrieux, Guus J. Heynen, Stefan Fichtner-Feigl, Luciana Hannibal, Dietrich A. Ruess

**Affiliations:** 1 Department of General and Visceral Surgery, Center for Surgery, Faculty of Medicine, Medical Center – University of Freiburg, Freiburg, Germany; 2 Faculty of Biology, University of Freiburg, Freiburg, Germany; 3 Department of Hepatobiliary and Pancreatic Surgery, The Affiliated Cancer Hospital of Zhengzhou University & Henan Cancer Hospital, Zhengzhou, China; 4 Institute of Neuropathology, Faculty of Medicine, Medical Center – University of Freiburg, Freiburg, Germany; 5 Institute of Medical Bioinformatics and Systems Medicine, Faculty of Medicine, Medical Center – University of Freiburg, Freiburg, Germany; 6 Institute of Surgical Pathology, Faculty of Medicine, Medical Center – University of Freiburg, Freiburg, Germany; 7 Division of Hematology, University Hospital Basel, Basel, Switzerland; 8 Department of Internal Medicine II, Medical Center – University of Freiburg, Freiburg, Germany; 9 CIBSS – Centre for Integrative Biological Signalling Studies, University of Freiburg, Freiburg, Germany; 10 German Cancer Consortium (DKTK), Partner site Freiburg, a partnership between DKFZ and Medical Center – University of Freiburg, Freiburg, Germany; 11 Department of Medicine I, Faculty of Medicine, Medical Center – University of Freiburg, Freiburg, Germany; 12 Faculty of Medicine, Department of Neuroanatomy – University of Freiburg, Freiburg, Germany; 13 Division of Medical Physics, Department of Diagnostic and Interventional Radiology, Faculty of Medicine, University Medical Center Freiburg, University of Freiburg, Freiburg, Germany; 14 Berlin Center for Advanced Therapies (BeCAT), Charité - Universitätsmedizin Berlin, Berlin, Germany; 15 Department of Pediatrics, Faculty of Medicine, Medical Center – University of Freiburg, Freiburg, Germany

**Keywords:** Cancer metabolism, Cancer therapy

## Abstract

Oncogenic KRAS mutations drive metabolic reprogramming in pancreatic ductal adenocarcinoma (PDAC). Src-homology 2 domain-containing phosphatase 2 (SHP2) is essential for full KRAS activity, and promising dual SHP2/mitogen-activated protein kinase (MAPK) inhibition is currently being tested in clinical trials. Exploitable metabolic adaptations may contribute to invariably evolving resistance. To understand the metabolic changes induced by dual inhibition, we comprehensively tested human and murine PDAC cell lines, endogenous tumor models, and patient-derived organoids, which are representative of the full spectrum of PDAC molecular subtypes. We found that dual SHP2/mitogen-activated protein kinase kinase (MEK1/2) inhibition induces major alterations in mitochondrial mass and function, impacts reactive oxygen species (ROS) homeostasis and triggers lipid peroxidase dependency. Anabolic pathways, autophagy and glycolysis were also profoundly altered. However, most strikingly, mitochondrial remodeling was evident, persisting into a therapy-resistant state. The resulting vulnerability to the induction of ferroptotic cell death via the combination of vertical SHP2/MEK1/2 with glutathione peroxidase (GPX4) inhibition was largely independent of the PDAC molecular subtype and was confirmed with direct targeting of RAS. The triple combination of SHP2/MEK1/2 inhibition and the ferroptosis-inducing natural compound withaferin A suppressed tumor progression in an endogenous PDAC tumor model in vivo. Our study offers a metabolic leverage point to reinforce RAS pathway interference for targeted PDAC treatment.

## Introduction

Pancreatic ductal adenocarcinoma (PDAC) remains one of the deadliest cancers, with an estimated rise to the second leading cause of cancer-related deaths in the US within the coming years.^1^ Despite recent therapeutic advances, patients with PDAC still have a poor prognosis, with more than 85% of patients succumbing to the disease within 5 years.^2^ Surgery is the only curative option, but only approximately 20% of cases are upfront resectable, and recurrence is common even after adjuvant chemotherapy.^3^ The genetic landscape of PDAC has been extensively studied, and oncogenic KRAS mutations are clearly the dominant drivers of this entity, with more than 90% of PDAC tumors displaying activating alterations in the *KRAS* gene.^4^ KRAS wild-type (WT) PDAC frequently harbors genetic alterations in other RAS pathway genes,^4^ confirming the high level of dependence of this entity on this signaling cascade. KRAS drives a variety of cellular functions, including cell proliferation, differentiation, survival, and, importantly, metabolic adaptation.^5–7^

PDAC can be subclassified on the basis of clinically relevant transcription profiles.^8–11^ PDAC patients with tumors with a more dedifferentiated and mesenchymal expression pattern (basal-like/quasimesenchymal/squamous subtypes) appear to have a distinctly worse prognosis than patients with tumors with a differentiated, epithelial expression pattern (classical/progenitor subtypes).^8–12^ Initially, epithelial subtypes were found to correlate with KRAS dependency^13,14^; more recently, an increase in the gene dosage of mutated *KRAS* was described to directly impact PDAC biology and determine a more dedifferentiated and more aggressive phenotype.^9,15,16^

Furthermore, three metabolic subtypes of PDAC have been described, differing mainly in their dependence on glycolytic, lipogenic, and redox pathways, with a suggested correlation with transcriptional signatures.^17^ Specifically, the glycolytic subtype tends to align with the basal-like transcriptional signature, whereas the lipogenic subtype is more strongly associated with the classical transcriptional profile.^17^

A major hallmark of cancer cells is their ability to reprogram metabolism to sustain the energetic and biosynthetic demands of continuous proliferation.^18^ In PDAC, mutated KRAS has been identified as a key driver of metabolic reprogramming.^19,20^ Notably, mutant KRAS^G12D^ has been shown to increase glycolysis, which, for example, fuels ribose biogenesis through increased flux into the nonoxidative pentose phosphate pathway.^19^

Consistent with this, glycolysis-related transporters such as monocarboxylate transporters 1 and 4 (MCT1 and MCT4), as well as the rate-limiting enzymes hexokinase 1 and 2 (HK1 and HK2), are upregulated in PDAC, thereby supporting increased glycolytic flux.^20,21^

Next to glycolysis, PDAC cells also exhibit profound adaptations in oxidative phosphorylation (OXPHOS) and redox homeostasis. KRAS has been shown to drive a metabolic cascade via glutamine-derived aspartate conversion through aspartate transaminase (GOT1), leading to an increased NADPH/NADP+ ratio that helps maintain redox balance in favor of tumor survival.^22^ Furthermore, oncogenic KRAS can activate the NRF2 antioxidant system, further supporting redox homeostasis.^23^ In addition, autophagy, a lysosome-mediated recycling process, is upregulated in KRAS-mutant PDAC, and the suppression of KRAS further elevates the already high basal autophagy levels.^24,25^

Previous studies have demonstrated that dual “vertical” RAS pathway inhibition by targeting Src homology region 2 domain-containing phosphatase 2 (SHP2) and mitogen-activated protein kinase kinase (MAP2K1/2) MEK1/2 or extracellular signaling kinases (MAPK3/1) ERK1/2 leads to synergistic growth inhibition of PDAC tumors.^26,27^ Clinical trials (e.g., NCT04916236) are currently underway to explore the potential of this approach for patients. Additionally, the rapidly expanding field of direct KRAS and RAS inhibition has already had a tremendous impact.^28–32^ However, as with MEK or ERK inhibitors, quickly evolving therapy resistance needs to be anticipated, and combination strategies will likely be required to achieve durable responses. Cotargeting SHP2 is promising in this context.^33^

Here, we aimed to uncover therapeutically exploitable PDAC subtype-dependent metabolic vulnerabilities that evolve with indirect dual interference with RAS pathway activity via vertically combined SHP2/MEK1/2 inhibition.

## Results

### In vitro screening reveals that mitochondrial respiration is a common dependency in response to dual SHP2/MEK inhibition

**Fig. 1:**
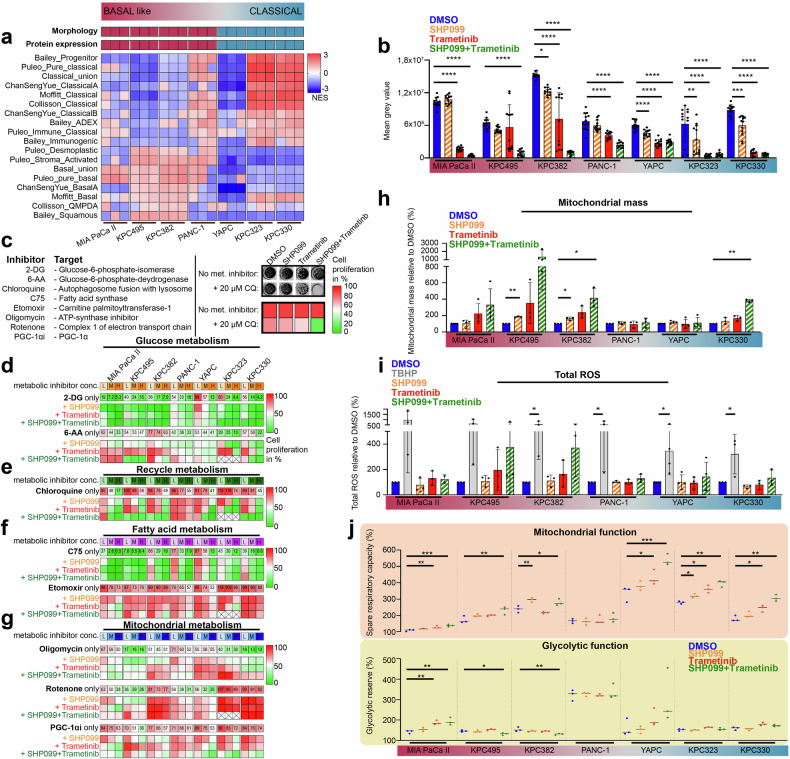
Pharmacological SHP2 and/or MEK inhibition reprograms PDAC cell metabolism. **a** Transcriptomic classification of human and murine PDAC cell lines into a spectrum of basal-like to classical subtypes. **b** Proliferation of PDAC cell lines treated with DMSO, SHP099 (15 μM), trametinib (10 nM), or both. The data represent the SDs from 12 wells in one experiment. **c** *Left*: Metabolic inhibitors combined with MAPK pathway inhibition. *Right*: Example of a proliferation assay showing color-coded MAPK treatment comparisons at ~90% confluency, with or without metabolic inhibitors. **d**–**g** Relative cell proliferation under MAPK pathway inhibition with low (L), medium (M), and high (H) metabolic inhibitor concentrations in PDAC cells. The heatmap represents the mean of eight wells across two independent experiments; the metabolic inhibitor concentrations are depicted in Supplementary Fig. 2. For the crossed-out areas, no data could be obtained. **h** Mitochondrial mass in PDAC cells was measured via flow cytometry, and three to four independent experiments were performed. **i** Intracellular ROS by flow cytometry, with tert-butyl hydroperoxide (TBHP) as the ROS-inducing control; mean of two to four independent experiments. **j** Spare respiratory capacity and glycolytic reserve in PDAC cells, as assessed via the ECAR and OCR in three experiments with six technical replicates each. Statistical significance was determined via one-way ANOVA in (**b**, **h**, **i**, **j**), with comparisons made against corresponding DMSO controls. *P < 0.05, **P < 0.01, ***P < 0.001, ****P < 0.0001

We selected a panel of PDAC cell lines to screen for metabolism-associated subtype-specific vulnerabilities upon SHP2/MEK inhibition in vitro. The human cell lines MIA PaCa II [KRAS^G12C^, TP53^R248W^], PANC-1 [KRAS^G12D^, TP53^R273H^], and YAPC [KRAS^G12V^, TP53^H179R^] were utilized, as were a number of murine cell lines established from endogenous genetic tumors of the autochthonous *Kras*^LSL-G12D/+^; *Trp53*^fl/fl^; *Ptf1a*^Cre-ex1/+^ (KPC) PDAC model. The cell lines were classified and chosen according to their molecular subtype on the basis of morphology, protein expression profile, and transcriptomic gene set enrichment to guarantee representative, cross-species coverage of the basal-to-classical PDAC subtype spectrum (Fig. 1a and Supplementary Fig. 1a–e).

The allosteric SHP2 inhibitor SHP099 had only a minimal effect on proliferation, while the MEK1/2 inhibitor trametinib separated the cell lines into primary sensitive (MIA PaCa II, KPC323, KPC330) and intrinsic/rapid-adaptive resistant (KPC382, KPC495, PANC-1, and YAPC) cell lines (Fig. 1b). Combined SHP099/trametinib therapy increased sensitivity or overcame intrinsic resistance, with no subtype-specific response observed, suggesting broad applicability and therapeutic value for dual SHP2/MEK inhibition (Fig. 1b).

To gain insight into potential dependencies on specific metabolic pathways, the cell lines were then exposed to various metabolic inhibitors with or without MAPK pathway inhibition (Fig. 1c, left). We allowed each MAPK treatment setup (without a metabolic inhibitor) to reach full confluency and then compared the area covered with the same treatment plus the metabolic inhibitor. This normalization enabled us to isolate the specific impact of metabolic inhibition in a prolonged culture setting of 6–14 days (exemplified in Fig. 1c, right).

Glycolysis inhibition via 2-deoxy-D-glucose (2-DG) indicated higher sensitivity for basal-like cell lines. Adding SHP2 and/or MEK inhibition further sensitized classical murine cell lines without altering the high sensitivity of basal-like lines (Fig. 1d, top panel; Supplementary Fig. 2).

Pentose phosphate pathway (PPP) inhibition via 6-aminonicotinamide (6-AA) had heterogeneous results, with either increased or decreased PPP dependency across subtypes (Fig. 1d, bottom panel; Supplementary Fig. 2). The dependency of PDAC on autophagy, particularly after MAPK pathway inhibition, was confirmed in 6 out of 7 cell lines via the use of chloroquine (CQ). Compared with trametinib alone, dual SHP2/MEK inhibition further potentiated this effect, increasing CQ sensitivity, irrespective of the molecular subtype (Fig. 1e, Supplementary Fig. 2). Fatty acid synthase (FAS) inhibition with C75 impaired proliferation across all the cell lines but did not affect SHP2 or MEK inhibition (Fig. 1f, Supplementary Fig. 2). However, fatty acid oxidation (FAO) interference via etomoxir increased sensitivity predominantly in rather basal-like cell lines (MIA PaCa II, KPC495, KPC382, and PANC-1), although off-target mitochondrial effects from etomoxir concentrations^34^ might have influenced the results.

Further screening revealed changes in mitochondrial respiration, particularly with dual SHP2/MEK inhibition (Fig. 1g, top panel, Supplementary Fig. 2). Trametinib dampened ATP-synthase inhibitor oligomycin-induced proliferation impairment in 2/3 classical and 2/3 basal-like lines, with a more pronounced effect under combination therapy. Similar patterns were obtained with rotenone, a complex 1 inhibitor, whose potency was attenuated by the addition of trametinib in 3/3 of the basal-like lines and in 1/3 of the classical lines, and even more so with dual inhibition (Fig. 1g, middle panel, Supplementary Fig. 2). PGC-1α inhibition (PGC-1αi) increased sensitivity to dual inhibition across subtypes, further supporting a central role of mitochondrial metabolism (Fig. 1g, bottom panel, Supplementary Fig. 2).

Given the screening data indicating that mitochondria are a dominant metabolic target in response to dual SHP2/MEK inhibition across PDAC subtypes, we investigated mitochondrial behavior under MAPK pathway inhibition in more detail. Dual SHP099/trametinib therapy increased the mitochondrial mass in both the basal-like and classical cell lines, whereas the reactive oxygen species (ROS) levels tended to increase (Fig. 1h, i). Electron microscopy revealed changes in the number and microstructure of mitochondria, particularly in response to combination therapy, but a fully consistent pattern was not detected (Supplementary Fig. 3a, b). To gain insight into mitochondrial respiratory function, oxygen and proton flux analysis via Seahorse assays was subsequently performed. Dual SHP2/MEK inhibition significantly increased spare respiratory capacity in most cell lines, as indicated by the ratio of basal respiration to maximum respiration, suggesting improved mitochondrial fitness (Fig. 1j, top panel). Basal respiration decreased in 2/3 of the basal-like lines (Supplementary Fig. 4a, top), whereas maximal respiration increased notably in the classical lines (Supplementary Fig. 4a, middle), likely driving increased respiratory capacity in the classical group. Proton leakage analysis revealed no notable differences in oxidative phosphorylation (OXPHOS) efficiency between cell lines or treatments (Supplementary Fig. 4a, bottom). As mutated KRAS has been shown to drive glycolysis, we assessed this metabolic process under MAPK pathway inhibition. Seahorse assays revealed no significant differences in the glycolytic reserve (Fig. 1j, bottom) or basal glycolysis between basal-like and classical cells (Supplementary Fig. 4b).

Taken together, these in vitro data suggest that indirect interference with RAS activity via dual SHP2/MEK inhibition leads to substantial metabolic changes in PDAC tumor cells. While increased dependence on autophagy is further amplified in comparison with MEK inhibition alone, metabolic alterations are particularly linked to changes in mitochondrial abundance and function across molecular subtypes.

### Multiomics confirm mitochondrial adaptations in response to SHP2/MEK inhibition

To investigate the underlying properties of the screening response of our cell lines, we performed metabolomic, proteomic, and transcriptomic analyses. The human cell lines MIA PaCa II, PANC-1, and YAPC were subjected to mass spectrometric analysis of intra/extracellular metabolites and targeted proteomics.

**Fig. 2:**
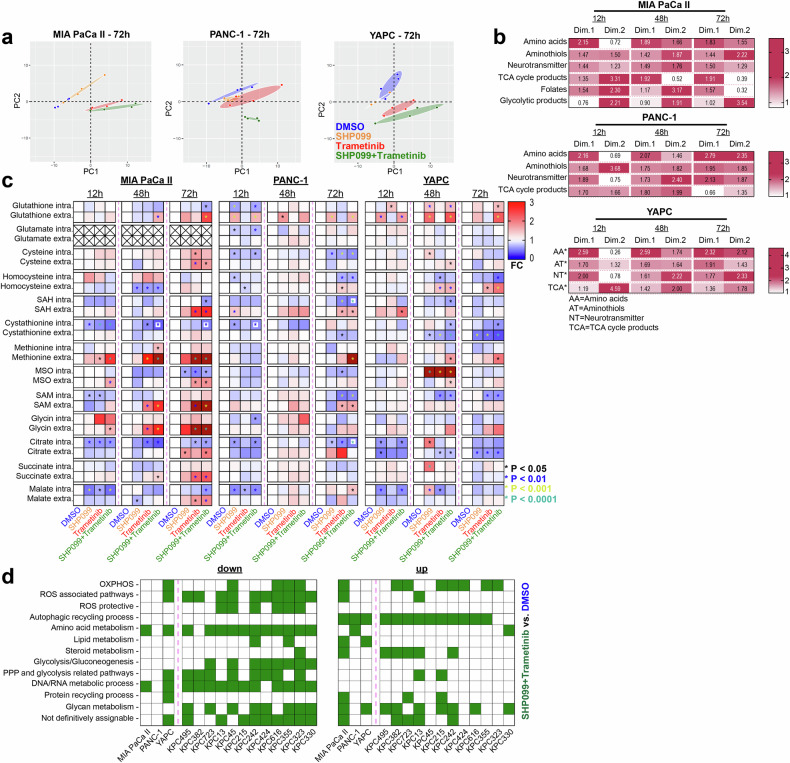
Pharmacological SHP2 and/or MEK inhibition reshapes both the intra- and extracellular metabolite profiles. **a** PCA of extracellular metabolites in PDAC cell lines treated for 72 h with DMSO, SHP099 (15 μM), trametinib (10 nM), or the combination (four samples per treatment; 62 metabolites for MIA PaCa II, 54 for PANC-1 and YAPC). **b** Summarized PCA contribution factors that indicate how much each variable influences the principal components. PCA contribution of human PDAC cell lines at 12, 48, and 72 h of treatment, showing variable loadings on the first two principal components, which capture the most variance. The values for the various metabolic pathways (e.g., amino acids) represent the means of the individual metabolites shown in Supplementary Fig. 5. **c** Heatmap of intra- and extracellular metabolite levels in cells treated with SHP099, trametinib, or both vs. DMSO controls (set to 1). The color gradient shows the fold changes, with dark red indicating values > 3 for clarity. For the crossed-out areas, no data could be obtained. **d** KEGG-based gene set enrichment analysis (GSEA) was performed on human and murine PDAC cells treated with DMSO or the combination of SHP099 and trametinib for 48 h. Each colored square represents a significant upregulation or downregulation of at least one gene set within a metabolic category relative to DMSO, with the individual gene sets listed in Supplementary Fig. 7b. “Not definitively assignable” indicates unclear metabolic classification. Gene sets with an adj. *P* < 0.25 are shown. Statistical significance was determined via one-way ANOVA in (**c**), with comparisons made against corresponding DMSO controls. * *P* < 0.05, ** *P* < 0.01, *** *P* < 0.001, **** *P* < 0.0001

Metabolite analysis of the extracellular compartment revealed substantial alterations following therapy (Fig. 2a, Supplementary Fig. 5a–c), particularly after 72 h, and most prominently with SHP2/MEK inhibition. Amino acids were the primary contributors to distinct metabolite profiles across treatments (Fig. 2b, Supplementary Fig. 5d–f), with dual SHP2/MEK inhibition therapy significantly increasing extracellular amino acid levels (Supplementary Fig. 6a, data for other cell lines not shown). Time-course analysis suggested that these elevated levels arose from reduced uptake rather than increased secretion, as shown by serine and valine in MIA PaCa II cells (Supplementary Fig. 6b). An increased abundance of amino thiols and neurotransmitters was also observed in the extracellular milieu (Fig. 2b, Supplementary Fig. 5d–f). Given the pronounced mitochondrial alterations under dual SHP2/MEK inhibition therapy, we focused on metabolites linked to mitochondrial and ROS metabolism. In MIA PaCa II cells, the levels of glutathione, S-adenosylhomocysteine (SAH), cystathionine, methionine sulfoxide (MSO), malate, and citrate decreased intracellularly but increased extracellularly with combination therapy (Fig. 2c). Similar trends were observed in PANC-1 and YAPC cells, although these trends were less pronounced. Lipid metabolism also shifted, with an increase in acylcarnitine in MIA PaCa II cells across all treatments, including SHP099 monotherapy (Supplementary Fig. 6c).

Targeted proteomics revealed minimal effects of SHP099 monotherapy (Supplementary Fig. 6d–f), whereas trametinib had a stronger effect, which was further pronounced with dual SHP2/MEK inhibition. Proteins involved in mitochondrial metabolism increased in MIA PaCa II and PANC-1 cells following combination therapy (Supplementary Fig. 6g), and the expression of mitochondrial fusion/fission proteins increased. Ferroptosis regulators also changed: GPX4 expression was downregulated in PANC-1 and YAPC cells under trametinib and combination therapy. Other ferroptosis-related proteins, such as decreased ferroptosis suppressor protein 1 (FSP1) in MIA PaCa II and YAPC and increased long-chain-fatty-acid-CoA ligase 4 and transferrin receptor protein 1 in YAPC, exhibited notable changes. Collectively, metabolomics and proteomics confirmed that dual SHP2/MEK inhibition affects proliferation-related pathways, mitochondrial dynamics, and ferroptosis regulation.

For RNA sequencing, we integrated KPC mouse cell lines, expanding the screening collection with additional lines to ensure dense representation of murine PDAC subtypes (Supplementary Fig. 1a, c, e). SHP099 monotherapy minimally affected gene expression (Supplementary Fig. 7a), whereas trametinib and combination therapy had greater effects. Gene set enrichment analysis (GSEA) highlighted metabolic gene alterations (Fig. 2d and Supplementary Fig. 7b), revealing the downregulation of DNA/RNA and amino acid metabolism genes. Combined SHP2 and MEK inhibition most prominently upregulated lipid and steroid metabolism-related genes. The downregulation of glycolysis-related gene sets under trametinib and combination therapy confirmed the dependency of the cells on KRAS, whereas SHP2 inhibition had little effect. Compared with those with trametinib alone, autophagy-related gene sets were upregulated with combination therapy in nearly all the cell lines (Fig. 2d and Supplementary Fig. 7b). Significant changes in OXPHOS and ROS-associated pathways were also observed, particularly under combination therapy. ROS-protective gene sets, including those related to glutathione and cysteine/methionine metabolism, were downregulated in most murine cell lines treated with combination therapy (combined comparisons with DMSO and trametinib).

In summary, RNA sequencing analysis corroborated our functional observations regarding anabolism, glycolysis, autophagy, and mitochondrial function, without a clear correlation with molecular PDAC subtypes.

### Genetic knockout of SHP2 has a profound impact but does not recapitulate or provide information about pharmacologic SHP2 inhibition

To better understand the role of SHP2 in PDAC metabolism, we generated *PTPN11* knockout (KO) cell lines via CRISPR-Cas9 in human cell lines and employed murine cell lines derived from a dual recombinase PDAC model.^26^

KO was confirmed by Western blot and led to changes in KRAS and phospho-protein levels (pERK, pAKT, and/or pSTAT3) with individual patterns in different cell lines, indicating potential activation of different compensatory signaling pathways (Supplementary Fig. 8a). However, in most instances, the proliferation of KO cells was more severely impaired than that of SHP099-treated WT cells (Supplementary Fig. 8b).

Metabolically, SHP2 KO cells behaved distinctly from parental WT and SHP099-treated WT cells. MIA PaCa II KOs were more sensitive to OXPHOS and FAO inhibition and presented greater mitochondrial mass and respiration but reduced ROS (Supplementary Fig. 8c–e, g). This finding resembled the metabolic profile under dual SHP2 and MEK inhibition (Fig. 1g, h). KO cells also presented distinct extracellular metabolite profiles (Supplementary Fig. 8f) and transcriptional subtype shifts (Supplementary Fig. 8h). Murine KOs (F2453 and F2683) were largely similar, with F2683 showing greater compensation (Supplementary Fig. 8i–k).

In summary, genetic loss of *PTPN11* caused deeper metabolic and signaling rewiring than pharmacologic inhibition did, likely due to clonal selection and/or the complete loss of phosphatase-independent molecular adapter functions of SHP2, and may not reflect the phenotype induced by allosteric inhibition.

### Mitochondrial reprogramming is maintained in therapy-resistant cell lines

To study therapeutic resistance, the cell lines were treated with SHP099, trametinib, or both for ≥8 weeks, which induced adaptive resistance. The majority of the cell lines upregulated E2F targets, G2M checkpoints, or IL6-JAK-STAT3 responses. In particular, the human cell lines upregulated KRAS signaling, and the murine cell lines MYC targets (Supplementary Fig. 9a). The increase in mitochondrial mass observed with short-term treatment (Fig. 1h) was also observed in 2/3 of the basal-like lines after long-term exposure, whereas the classical lines presented a decrease (Supplementary Fig. 9b). The ROS levels increased notably in MIA PaCa II but remained unchanged in the other lines (Supplementary Fig. 9c).

Despite resistance, sensitivity to glycolysis inhibition remained largely unchanged, whereas PPP dependency increased, as reflected by increased 6-AA sensitivity in 6/7 resistant lines (Supplementary Fig. 9d, Fig. 1d). The sensitivity of 5/7 lines to autophagy inhibition by chloroquine persisted (Supplementary Fig. 9e, Fig. 1e). FAS inhibition via C75 had no additional effect, whereas etomoxir sensitivity increased in 2/7 of the trametinib- or combination-resistant lines (Supplementary Fig. 9f). Most resistant cell lines (trametinib: 5/7; combination: 4/7) presented increased sensitivity to rotenone and oligomycin (Supplementary Fig. 9g).

Interestingly, long-term treatment downregulated OXPHOS gene expression in all three human cell lines. Furthermore, MIA PaCa II, KPC323, and KPC330 downregulated ROS-protective pathways (Supplementary Fig. 9h).

Together, these data indicate a sustained impact of SHP2/MEK inhibition on mitochondrial metabolism in therapy-resistant states.

### Adaptive mitochondrial remodeling following dual SHP2/MEK inhibition is confirmed in vivo

**Fig. 3:**
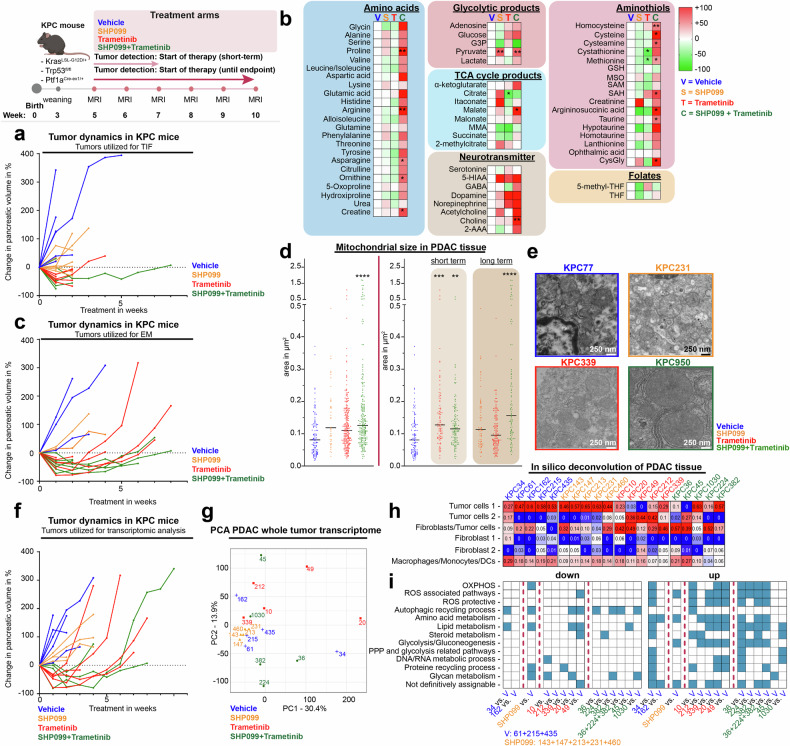
Dual SHP2 and MEK inhibition causes mitochondrial adaptations in vivo. The treatment schedule for the KPC mice is shown at the top left. The mice were treated with vehicle (control), SHP099 (75 mg/kg), trametinib (1 mg/kg), or their combination every other day. The mouse icon was sourced from BioRender.com. **a** Dynamics of KPC tumors in mice, from which tumor interstitial fluid was collected for analysis via LC‒MS/MS. **b** Metabolite abundance under targeted therapy relative to that in vehicle-treated tumors, organized by metabolic pathways. Supplementary Fig. 11 details individual metabolites. **c** Tumor dynamics in KPC mice, with tumors processed for electron microscopy. **d** Left: Mitochondrial diameter in PDAC tumor cells from KPC mice, shown for all therapy arms. Each dot represents a single mitochondrion. Right: Same data separated by short (≤2 weeks) and long (>2 weeks) therapy durations. **e** Representative mitochondrial morphology in KPC-PDAC tumor cells under targeted therapy. **f** Tumor dynamics in KPC mice, with tumors processed for whole-tissue RNA sequencing. **g** PCA of KPC tumor transcriptomes following therapy. **h** In silico deconvolution of RNA-sequenced KPC tumors. **i** KEGG-based GSEA of KPC tumor transcriptomes. Each colored square represents a significant up- or down regulation of at least one gene set within a metabolic category relative to the vehicle reference, with the individual gene sets listed in Supplementary Fig. 7b. Gene sets with an adj. *P* < 0.25 are shown. Statistical significance in (**b**, **d**) was determined by one-way ANOVA against vehicle controls: *P < 0.05, **P < 0.01, ***P < 0.001, ****P < 0.0001. G3P glyceraldehyde-3-phosphate, MMA methylmalonic acid, 5-HIAA 5-hydroxyindoleacetic acid, 2-AAA 2-aminoadipic acid, GSH glutathione, MSO methionine sulfoximine, SAM S-adenosylmethionine, SAH S-adenosylhomocysteine, CysGly cysteinylglycine, THF tetrahydrofolate

To determine whether the observed metabolic properties following SHP2/MEK inhibition prove true in vivo, we treated KPC mice, monitored the pancreatic tumor volume via magnetic resonance imaging (MRI), and collected tumor interstitial fluid (TIF). As illustrated in the treatment overview at the top of Fig. 3, animals were assigned to either a short-term cohort (reaching their endpoint after a 2-week treatment interval) or a survival cohort, in which treatment was continued until the respective survival endpoint and animals were scarified upon severe morbidity. This approach allowed us to capture a broad range of treatment responses. The tumors used for TIF isolation resembled the previously described^26^ pattern of response to SHP2/MEK inhibition in terms of tumor dynamics (Fig. 3a).

Metabolite profiling of TIFs via LC‒MS/MS revealed no significant differences in protein concentration (Supplementary Fig. 10a). While principal component analysis (PCA) of the full sample set did not show clear clustering by treatment, three combination-treated tumors formed a distinct subcluster, which was correlated with tumor volume reduction (Supplementary Fig. 10b, c). The other two combination samples were either treated for a prolonged period (55 days) or did not respond to the treatment in the same manner.

To maintain consistency, we selected TIFs from tumors that exhibited volume regression during 2 weeks of therapy for further analysis (Supplementary Fig. 10c). This refined PCA revealed separate clustering for combination-treated TIFs (Supplementary Fig. 10d). Metabolite analysis revealed elevated amino acid levels in combination-treated tumors, which was consistent with reduced uptake due to proliferation inhibition (Fig. 3b, Supplementary Fig. 11). Glucose and glyceraldehyde-3-phosphate levels suggested reduced glycolysis in both the trametinib and combination therapy groups (Fig. 3b, Supplementary Fig. 11). TCA cycle intermediates such as alpha-ketoglutarate and citrate behaved similarly between treatments, although malate was significantly increased in the interstitial space of the combination therapy samples (Fig. 3b, Supplementary Fig. 11).

Interestingly, an increased abundance of neurotransmitters was detected, particularly in TIFs from SHP099/trametinib-treated tumors (Fig. 3b, Supplementary Fig. 11).

Finally, the composition of the amino thiols in the TIFs indicated changes in mitochondrial respiration and ROS defense. A comparison of SHP099 and trametinib with combination treatment revealed striking differences; for example, combination treatment resulted in significantly elevated levels of cysteine, methionine, S-adenosylmethionine (SAM), and SAH (Fig. 3b, Supplementary Fig. 11). The upregulation of the methionine cycle and transsulfuration pathway may be elicited to counter the anti-proliferative and pro-oxidative effects of the combined treatment, respectively.

In summary, compared with those treated with trametinib alone, KPC tumors treated with dual SHP099/trametinib therapy presented distinct metabolic changes in the interstitial space, particularly in terms of mitochondrial respiration and ROS protection.

To further substantiate our observations, we treated KPC mice and collected fresh samples after short-term and survival endpoint treatments, confirming tumor dynamics (Fig. 3c). Electron microscopy revealed significantly larger mitochondria in tumor cells from tumors treated with SHP099/trametinib than in those from controls (Fig. 3d, left). Both short-term treatments increased the mitochondrial size, but only SHP099/trametinib had a persistent effect (Fig. 3d, right). Additionally, the mitochondrial cristae were more densely packed, with intra- and intertumoral heterogeneity (Fig. 3e). These results align with our in vitro observations, reinforcing the conclusion that dual SHP2 and MEK inhibition induces significant mitochondrial alterations in PDAC both in vitro and in vivo.

We then performed bulk RNA sequencing of tumor tissue from animals treated until the survival endpoint (Fig. 3f). PCA revealed distinct transcriptomic profiles, demonstrating intertumoral heterogeneity, with three vehicle-treated samples (KPC61, KPC215, and KPC435) clustering together as a reference for comparison (Fig. 3g). In silico deconvolution indicated that epithelial cells dominated the bulk-seq transcripts, followed by fibroblasts and myeloid cells (Fig. 3h), supporting conclusions regarding mainly tumor cell transcription. GSEA revealed the upregulation of OXPHOS-related pathways, as well as ROS-associated pathways, in the trametinib-treated (3/5) and combination-treated (4/6) samples (Fig. 3i).

**Fig. 4:**
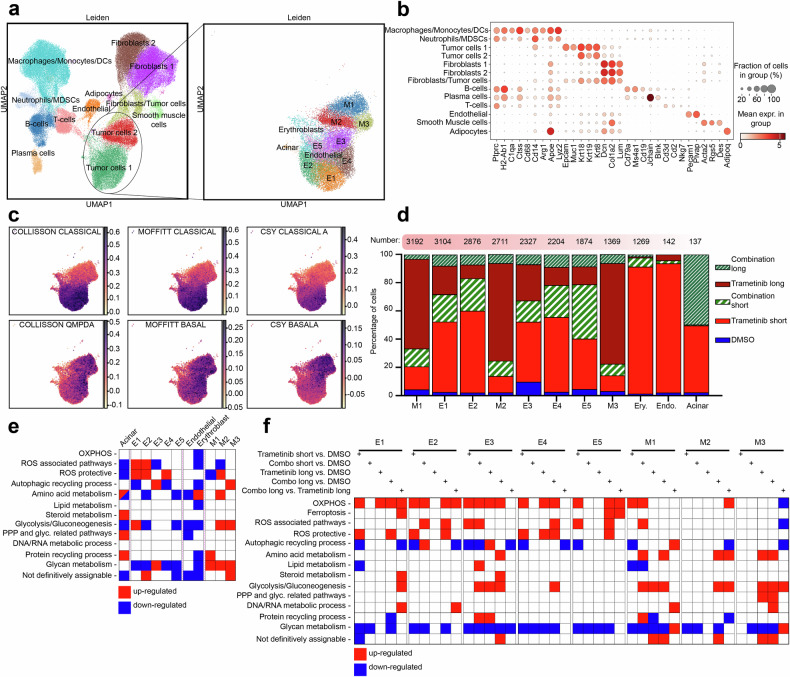
Mouse PDAC tissue single-cell transcriptomics confirm the involvement of mitochondria in the adaptive response to dual SHP2 and MEK inhibition. **a** Left: Batch-corrected UMAP plot of cells from PDAC tissue from individual KPC mice treated with vehicle (acting as a control), trametinib (1 mg/kg), or the combination of SHP099 (75 mg/kg) and trametinib. KPC mice treated for ≤2 weeks (short) or >5 weeks (long) were included. Right: UMAP plot highlighting the subclustering of the tumor cell/epithelial clusters. **b** Dot plot displaying the expression levels of selected genes across various cell types. **c** PDAC subtype signature enrichment and its contributions to the tumor cell/epithelial subclusters. Gene sets according to Collisson et al., Moffitt et al., and Chan-Seng-Yue (CSY) et al. were applied. **d** Percent distribution of the indicated treatments across different tumor cell/epithelial subclusters. **e** Cluster-specific gene set enrichment analysis within the tumor cell/epithelial compartment, including the top 500 up- and downregulated differentially expressed genes. **f** Treatment-specific GSEA within the tumor cell/epithelial compartment, based on the top 500 up- and downregulated DEGs. In (**e**, **f**), each colored square represents a significant up- or downregulation of at least one gene set within a metabolic category, with the individual gene sets listed in Supplementary Fig. 7b. Gene sets with an adj. *P* < 0.25 are shown. DCs dendritic cells, MDSCs myeloid-derived suppressor cells

To obtain more precise insight into cell-specific transcriptional alterations in treated KPC tumors, we conducted single-cell RNA sequencing (scRNA-seq) of tumors from treated KPC mice. Tumor cell clusters were identified and subclustered into groups with predominantly epithelial (E1–E5) and mesenchymal (M1–M3) features, corresponding to the classical and basal-like transcriptional subtypes, respectively (Fig. 4a–c). Notably, mesenchymal clusters (M1–M3) were enriched in tumors after long-term trametinib treatment but less so after long-term dual SHP099/trametinib treatment (Fig. 4d).

Metabolic gene set analysis revealed dominant alterations in ROS-associated pathways across the majority of the tumor cell clusters (Fig. 4e). Combination therapy affected the transcription of OXPHOS gene sets in most tumor cell clusters, alongside ROS-associated and ROS-protective pathways (Fig. 4f).

Overall, altered mitochondrial number and function were confirmed in vivo in PDAC tumor cells, particularly following dual SHP2/MEK inhibition. The data also indicate a sustained and even more pronounced effect in terms of mitochondrial adaptations with long-term treatment conditions under which therapy-resistant tumors have evolved.

### An exploitable dependency on GPX4 evolves with RAS pathway inhibition

As previously suggested by in vitro analyses (Figs. 1h, i and 2c and supplementary Fig. 6g), mitochondrial adaptations following dual SHP2/MEK inhibition appear to correlate with oxidative stress and lipid peroxidation within tumor cells. Lipid peroxidation refers to the oxidative degradation of polyunsaturated fatty acids within cellular membranes, leading to the formation of reactive aldehydes such as malondialdehyde (MDA). This process disrupts membrane integrity and is a key driver of ferroptosis, an iron-dependent form of regulated cell death characterized by the accumulation of lipid peroxides.

**Fig. 5:**
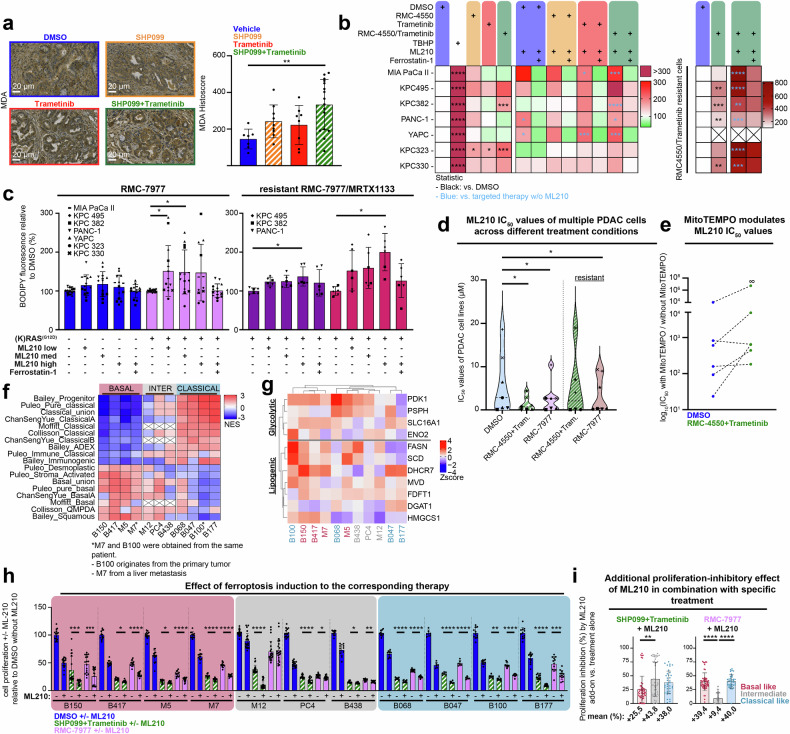
RAS pathway inhibition triggers lipid peroxidase dependency. **a** Left: Representative images of MDA staining. Right: Quantification of MDA levels in KPC tumors treated with vehicle, SHP099 (75 mg/kg), trametinib (1 mg/kg), or their combination. Each dot represents the histoscore of a single mouse, calculated as the mean of five to ten tumor regions analyzed per animal. **b** Heatmap showing relative lipid peroxidation (C11-BODIPY fluorescence) across multiple PDAC cell lines. The values are normalized to those of the DMSO control. TBHP served as a positive control, and ferrostatin-1 served as an antioxidant rescue control. The quantification results are shown in Supplementary Fig. 12b. Crossed-out areas indicate missing data. **c** Detection of lipid peroxidation via C11-BODIPY fluorescence in the context of RAS inhibition. Left: Effect of the addition of RMC-7977 (10 nM). Experiments were conducted in seven independent PDAC cell lines, each measured in two technical replicates per condition. Right: Lipid peroxidation in cells resistant to RMC-7977 (in purple, 10 nM) or MRTX1133 (in magenta, 10 µM). Experiments were conducted in three independent PDAC cell lines, each measured in two technical replicates per condition. ML210 concentrations were adapted to individual cell line sensitivity: low/medium/high concentrations - MIA PaCa II: 0.25/0.5/1 µM; KPC495: 5/10/20 µM; KPC382: 50/75/100 µM; PANC-1: 0.1/0.25/1 µM; YAPC: 10/15/20 µM; KPC323: 50/75/100 µM; KPC330: 10/20/40 µM. All values are shown relative to the corresponding treatment without ML210. **d** Determination of ML210 IC_50_ values across different treatment conditions in multiple PDAC cell lines. **e** ΔIC_50_ values for human and murine PDAC cell lines treated with DMSO or RMC-4550 + trametinib are shown, calculated as the IC_50_ in the presence of MitoTEMPO relative to the IC_50_ without MitoTEMPO. The values are plotted on a logarithmic scale. **f** Transcriptomic analysis classifying human PDAC organoids into basal-like or classical subtypes via ssGSEA. The color code represents the normalized enrichment score (NES) of each PDAC subtype signature. **g** Row-scaled intensity (*z*-score) of glycolytic and lipogenic genes across PDAC organoid samples. Rows and columns were hierarchically clustered via the complete linkage method with Euclidean distance as the similarity metric. **h** Relative proliferation of organoids treated with SHP099 (15 μM) + trametinib (10 nM) or RMC-7977 (10 nM) with or without ML210 (15 µM). The values are normalized to those of untreated controls. The data are based on two to four biological replicates with five technical replicates each. **i** Increased subtype-specific proliferation-inhibitory effects of ML210 in combination with the respective MAPK-pathway-targeted therapies. In (**b**) (left panel), statistical significance was assessed via one-way ANOVA, with black asterisks indicating differences between the untreated (DMSO) and treatment groups and blue asterisks denoting differences between the groups treated with or without ML210. Statistical comparisons with the DMSO-treated controls shown in (**c**, **d**) were also performed via one-way ANOVA. As shown in (**i**), one-way ANOVA was conducted to assess differences between subtypes in general. As shown in (**h**), differences between groups with and without ML210 were evaluated via unpaired *t*-tests. *P < 0.05, **P < 0.01, ***P < 0.001, ****P < 0.0001

To assess membrane lipid peroxidation in vivo, we performed MDA immunohistochemistry on KPC tumor tissue sections following treatment. Quantification via histoscore analysis revealed a significant increase in MDA staining in tumors treated with the combination therapy (Fig. 5a), indicating a cellular state with redox imbalance and a vulnerability to induce ferroptotic cell death via the addition of GPX4 inhibition.

To evaluate this observation in a broader and more controlled context, we systematically analyzed lipid peroxidation levels in our in vitro models. We treated various cell lines with the SHP2 inhibitor RMC-4550, trametinib, and the GPX4 inhibitor ML210. Owing to high autofluorescence interfering with certain assays, we used RMC-4550 instead of SHP099 in some of the following experiments. This decision was based purely on technical limitations and not on SHP099-specific toxicity or other biological effects. Importantly, the cellular response to RMC-4550 was comparable to that to SHP099 (Supplementary Fig. 12a).

Assessment of lipid peroxidation via the C11-BODIPY lipid peroxidation sensor revealed an increase in the C11-BODIPY signal following SHP2/MEK inhibition compared with that in the DMSO control group, which reached statistical significance in 2/7 of the cell lines (Fig. 5b, Supplementary Fig. 12b). The addition of ML210 to SHP2/MEK inhibitors significantly further amplified lipid peroxidation in 4/7 cell lines (indicated by blue stars). Notably, in RMC-4550/trametinib-resistant cells, ML210 treatment consistently induced a pronounced and significant increase in lipid peroxidation across all analyzed cell lines (Fig. 5b, right and supplementary Fig. 12b).

In light of the burgeoning field of direct RAS inhibition, we investigated whether treatment with a KRAS^G12D^ mutant-specific or a pan-RAS inhibitor would phenocopy dual SHP2/MEK results. Indeed, we observed that pan-RAS inhibition via the tricomplex on-state inhibitor RMC-7977 potentiated lipid peroxidation in response to GPX4 inhibition (Fig. 5c, left). Comparable effects were also observed in resistant cells (Fig. 5c, right), including those resistant to RMC-7977 and the KRAS^G12D^-specific off-state inhibitor MRTX1133.

These findings suggest that tumor cells subjected to dual SHP2/MEK or RAS inhibitors are more susceptible to GPX4 inhibition, potentially because of states with elevated levels of lipid peroxidation. In support of these findings, we found that the IC_50_ values for the GPX4 inhibitor ML210 were significantly lower in treated cells than in untreated cells (Fig. 5d and Supplementary Fig. 13a).

As our previous data indicated that therapy induced adaptive mitochondrial remodeling, particularly under combined SHP2 and MEK inhibition (Figs. 1h, j and 3d, e), we next sought to determine whether this altered mitochondrial function contributes to the observed phenotype, i.e., increased sensitivity to GPX4 inhibition. Interestingly, we found that the ΔIC_50_ between conditions with and without the mitochondrial ROS scavenger MitoTEMPO was less pronounced in untreated cells than in those receiving RAS pathway interference via dual SHP2/MEK or pan-RAS inhibition (Fig. 5e and Supplementary Fig. 13a, b), indicating that mitochondrial oxidative stress is a causative link.

To control for autophagy as a potential modulator of ferroptosis sensitivity under dual SHP2/MEK inhibition, we treated human and murine KPC cell lines with ML210 in the presence or absence of chloroquine (Supplementary Fig. 13c). Lipid peroxidation levels determined by C11-BODIPY assays did not significantly differ.

To further confirm the involvement of ferroptosis, we established efficient GPX4 knockdown within 72 h in two human and murine PDAC cell lines, as confirmed by Western blotting (Supplementary Fig. 13d). In all the models, dual SHP2/MEK inhibition or RAS inhibition increased lipid peroxidation, and GPX4 depletion frequently amplified this effect (Supplementary Fig. 13e), albeit to a less striking extent than did GPX4 inhibition, which was likely biased by the higher cell densities required for the knockdown experiments.

In summary, these findings suggest that RAS pathway inhibition sensitizes PDAC cells to processes consistent with ferroptosis, potentially facilitated by altered mitochondrial function and mitochondrial oxidative stress.

### Patient-derived PDAC organoids confirm that GPX4 dependency in response to RAS pathway inhibition

To corroborate our previous findings, we employed patient-derived PDAC organoids (PDOs) from our in-house reverse translational platform. A total of 11 PDO lines were selected, representing basal-like (*n* = 4), classical (*n* = 4), and intermediate (*n* = 3) transcriptional subtypes (Fig. 5f). While the morphological characteristics of the selected organoid lines appeared quite similar (Supplementary Fig. 14a), basal-like organoids, in comparison with their classical counterparts, presented transcriptional enrichment for epithelial-to-mesenchymal transition (EMT) and TGF-β signaling, along with downregulation of oxidative phosphorylation (OXPHOS) and fatty acid metabolism under treatment-naïve conditions (Supplementary Fig. 14b).

In addition to being used for transcriptional classification, the organoid lines were also stratified into metabolic subtypes on the basis of previously published criteria.^17^ Transcriptional metabolic profiling revealed substantial heterogeneity even within well-defined transcriptional subtype groups, and the transcriptional subtype did not clearly correlate with the metabolic subtype (Fig. 5g and Supplementary Fig. 14c). Interestingly, all organoid lines responded similarly well to SHP2 and MEK and best to SHP2/MEK inhibition, and their sensitivity to SHP2 monotherapy was generally greater than that of 2D cell lines (Supplementary Fig. 14d).

We performed a similar screening experiment with metabolic inhibitors as above for the 2D cell lines. Upon dual SHP2/MEK inhibition, some basal-like PDOs (e.g., B150 and B100) exhibited increased sensitivity to glycolysis inhibition via 2-DG. However, no consistent increase in autophagy dependence was observed under the combination treatment (Supplementary Fig. 14e). FAO inhibition with etomoxir was broadly effective in classical lines and approximately 50% of basal-like lines, whereas classical PDOs presented increased sensitivity to PGC-1α inhibition. In contrast, OXPHOS inhibition via rotenone or oligomycin elicited heterogeneous responses that were independent of the transcriptional subtype (Supplementary Fig. 14e). Notably, PDOs showed substantial variability across biological replicates, and owing to culture constraints, metabolic inhibitor screening was conducted at a fixed endpoint for all conditions. As a result, certain dynamic metabolic shifts may not be fully captured, as with the 2D cell line screen.

Nevertheless, owing to our previous observation that SHP2/MEK- and RAS-inhibited PDAC cells exhibit increased sensitivity to GPX4 inhibition, we next investigated this phenotype in PDOs (Fig. 5h and Supplementary Fig. 14f). Strikingly, nearly all the PDO lines demonstrated an additive response to GPX4 inhibition with ML210, both in the context of SHP2/MEK and RAS inhibition. This effect (mean range: 10–44% increase in response, Fig. 5i) was independent of the molecular subtype. However, when stratified by transcriptional subtype, intermediate organoid lines appeared to benefit the most from the addition of ML210 in the context of SHP2/MEK inhibition and basal-like and classical organoid lines in the context of pan-RAS inhibition (Fig. 5i and Supplementary Fig. 14f).

Taken together, the PDO results validate our 2D in vitro observations and underscore the therapeutic potential of combining GPX4 inhibition with SHP2/MEK or RAS inhibition.

### Combined SHP2/MEK/GPX4 inhibition delays tumor progression in vivo

**Fig. 6:**
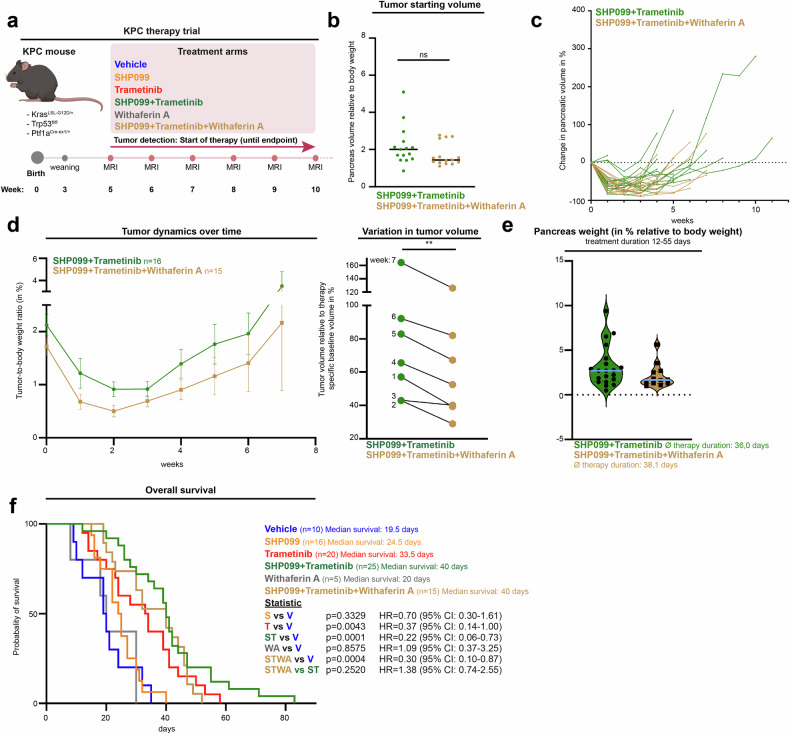
Combined SHP2/MEK/GPX4 inhibition delays tumor progression in vivo. **a** Schematic representation of the treatment trial with triple SHP099 (75 mg/kg) + trametinib (1 mg/kg) ± withaferin A (4 mg/kg) and the respective controls. Treatments were administered every other day. The mouse icon was sourced from BioRender.com. **b** Initial pancreatic volume relative to body weight before the start of treatment. **c** Individual volume changes over time relative to the baseline volume. **d** Left: Tumor dynamics over time are shown as the tumor-to-body weight ratio (%). The error bars indicate the standard error of the mean (SEM). Right: Pancreatic volumes at individual timepoints relative to the corresponding baseline volume. Each dot represents the mean tumor volume of multiple mice per treatment group and time point, normalized to the therapy-specific baseline volume at therapy initiation. **e** Endpoint pancreatic weights relative to body weight across treatment groups. **f** Kaplan‒Meier survival curves comparing treatment groups analyzed via the Mantel‒Cox log-rank test. Hazard ratios (HRs) and 95% confidence intervals (CIs) were calculated via the Mantel‒Haenszel method. For statistical analysis, unpaired two-tailed *t*-tests were performed in (**b**, **e**), whereas a paired two-tailed *t*-test was used in (**d**)

To translate our findings to an in vivo setting, we treated KPC mice with dual SHP2/MEK inhibition and additionally administered withaferin A (WA), a plant-derived compound previously reported to induce ferroptosis effectively in vivo.^35,36^ We first confirmed via C11-BODIPY assays that WA treatment is able to increase lipid peroxidation in vitro in both murine KPC and human PDAC cell lines (Supplementary Fig. 15a). WA was then administered to KPC mice either alone or concurrently with SHP2/MEK inhibition from the onset of treatment after tumor detection via MRI (Fig. 6a).

The pancreatic MRI volume at inclusion did not differ significantly from that of the reference cohort with dual SHP2/MEK inhibition only (Fig. 6b). Individual pancreatic volumes were then monitored longitudinally via weekly MRI until the survival endpoint of the study was reached (Fig. 6c and Supplementary Fig. 15b). WA monotherapy did not alter the course of the disease. However, tumor progression over time was significantly attenuated with the triple combination in comparison with dual SHP2/MEK inhibition (Fig. 6d). A potential reduction in final tumor weight was observed in the triple combination-treated group compared with the dual SHP2/MEK inhibition group, with a focus on animals with comparable survival times (Fig. 6e). MDA immunohistochemistry histoscore analysis revealed a marked increase in MDA staining with both dual SHP2/MEK inhibition and the addition of WA to SHP2/MEK inhibition relative to vehicle (Supplementary Fig. 15c). However, histoscores did not differ between the dual- and triple-therapy groups. Given the strong staining in both arms, we interpret the absence of a significant difference as saturation of in situ lipid peroxidation, not proving but also not excluding a further increase in ferroptotic cell death with triple therapy.

Animal body weight was monitored throughout the treatment period to evaluate the potential toxicity of the triple combination (Supplementary Fig. 15d). We did not observe any substantial weight loss over the course of triple treatment in comparison with dual SHP2/MEK inhibition. An increased incidence of ataxia as a termination criterion could be observed with SHP099/trametinib/WA (Supplementary Fig. 15e). As already observed with dual SHP2/MEK inhibition, most mice receiving the triple combination reached the humane endpoint owing to treatment-associated asthenia and not due to tumor burden. Consequently, the pronounced survival benefit achieved with dual SHP2/MEK inhibition could not be further extended (Fig. 6f). Notably, KPC mice are particularly small for age and fragile and exhibit limited physiological resilience, rendering them susceptible to intensified treatment regimens. Additionally, WA still has substantial limitations, and truly potent ferroptosis inducers with high-quality pharmacokinetic and pharmacodynamic properties remain to be identified.

Nevertheless, the results of this preclinical trial of endogenous murine PDAC corroborate our in vitro findings and support the concept that ferroptosis-inducing agents show promise for invigorating RAS pathway inhibition in patients with PDAC.

## Discussion

Even considering the burgeoning field of direct RAS inhibition, pancreatic adenocarcinoma (PDAC) remains among the cancers most difficult to treat. New orally bioavailable allosteric SHP2 inhibitors have demonstrated promising potential and have entered clinical evaluation for vertical RAS pathway inhibition combinations. While the addition of allosteric SHP2 inhibitors to MEK1/2-, ERK1/2-, or RAS-targeted drugs yields synergism and potently delays resistance development, therapy failure still needs to be anticipated and inadvertently occurs even with these combination strategies.

Previous studies have shown that oncogenic KRAS drives extensive metabolic rewiring in PDAC, including alterations in glycolytic flux^19,20^ and autophagy/mitophagy,^24,37^ as well as shifts in amino acid,^22^ lipid^38^ and cholesterol^39^ metabolism; nucleotide biosynthesis^40^; and impacts on mitochondrial function and redox homeostasis.^22,41^ This has been summarized in many excellent reviews, exemplified by Kerk et al.^42^ However, the precise metabolic consequences of vertical RAS pathway inhibition involving SHP2 remain uncharacterized. Here, we set out to uncover metabolic vulnerabilities induced by profound suppression of RAS-MAPK signaling through dual SHP2/MEK inhibition in PDAC, expanding on our prior findings.^26^ We employed PDAC cell lines, autochthonous in vivo models, and patient-derived organoids representing the full spectrum of molecular subtypes to reveal multilayered metabolic alterations in response to dual SHP2/MEK inhibition.

First, an overall reduction in glycolysis and/or PPP activity with MEK inhibition and SHP2/MEK combination therapy was observed, and virtually all the cell lines analyzed demonstrated sensitivity to glycolysis inhibition and corresponding transcriptomic alterations, highlighting the conserved dependency of PDAC on glycolytic pathways, which is consistent with the finding that KRAS signaling supports anabolic glucose metabolism.^19,20,41,43^ In this context, our data also indicate that basal-like cell lines rely on glycolysis even more strongly than classical cell lines do (Fig. 1d), confirming the literature linking the basal subtype to glycolytic metabolism in particular.^17^ Notably, the dependency on glycolysis persisted even in RAS pathway-inhibited resistant states.

While preclinical and early clinical studies have suggested that autophagy inhibition may enhance the therapeutic efficacy of MEK1/2 or ERK1/2 inhibition,^24,44^ real-life data have thus far not supported the reliable impact of trametinib + hydroxychloroquine for the treatment of metastatic PDAC.^45^ Our 2D in vitro data confirmed an increase in sensitivity to autophagy inhibition in the context of mono-MEK inhibition. This sensitivity was even further augmented with the addition of allosteric SHP2 inhibition, an effect that remained with long-term treatment. Transcriptomic data confirmed the upregulation of autophagy-related genes with dual SHP2/MEK inhibition therapy (Fig. 2d). These results indicate that dual SHP2/MEK in combination with autophagy inhibition, may be a more promising strategy for clinical application than MEK inhibition alone. SHP2 inhibitors may be of dual benefit here, since, notably, a recent study reported that allosteric SHP2 inhibitors could additionally inhibit autophagy as an off-target effect due to their accumulation in lysosomes.^46^ Our observation of inconsistent autophagy dependency in patient-derived organoids (PDOs) under vertical RAS pathway inhibition may be due to the shorter time spans of 3D culture conditions (only 72 h compared with 2D assays with 7–14 days, allowing compensatory mechanisms to become more relevant) and/or the lower proliferative activity and more complex architecture of PDOs, including 3D cell–cell interactions and oxygen/nutrient gradients, which may affect the regulation and necessity of autophagy differently than 2D cultures do.

Our results also revealed reduced DNA/RNA and amino acid metabolic processes in treated tumor cells (Figs. 2 and 3b). We interpreted these effects as closely linked to inhibited proliferation and consequently decreased anabolic processes. However, amino acid metabolism may influence the cellular state via reciprocal interactions with the tumor microenvironment (TME) in PDAC, with the involvement of cancer-associated fibroblasts and various immune cells. For example, alanine, which is produced by stromal fibroblasts, can serve as an important alternative carbon source for PDAC tumor cells,^47,48^ and neuron-derived serine may support PDAC growth in nutrient-poor environments.^49^ Furthermore, glutamine and cysteine uptake and metabolism have been demonstrated to maintain redox balance.^22,50–52^ Within the TME, elevated glutamine levels may support T-cell survival, whereas myeloid-derived suppressor cells (MDSCs) depend on glutamine to sustain their immunosuppressive functions, highlighting the metabolic complexity of cancer tissues.^53–55^

Crucially, our data demonstrate a pronounced impact of RAS pathway inhibition on mitochondrial dynamics both in vitro and in vivo, with the effects persisting into therapy-resistant states. Dual SHP2/MEK inhibition led to notable changes in mitochondrial metabolism (Figs. 1h, j, 2d and 3b, d, e). Tumor cells demonstrated mitochondrial remodeling in response to combination therapy, as indicated by increased mitochondrial mass and elevated ROS levels, especially in basal-like cell lines. Electron microscopy imaging and increased spare respiratory capacity supported these findings. Analyses of in vivo TIFs revealed significant differences in aminothiol levels between trametinib and combination therapy. Additionally, cysteine and methionine metabolism were downregulated in murine KPC cell lines (Fig. 2d). These alterations result in a dependency on lipid peroxidase pathways and increased vulnerability to the induction of ferroptotic cell death. Targeting GPX4 efficiently reinforces RAS pathway inhibition via vertical SHP2/MEK combination or (K) RAS directly. These observations are further supported by recent findings, indicating that ROS-associated pathways and ferroptosis protection mechanisms play important roles in late-stage adaptive resistance in cancer cells at the end of the adaptive resistance continuum.^56^

Our findings build on two previously reported PDAC-specific characteristics, namely, 1st RAS signaling driving glutamine metabolism for ROS homeostasis^22,50,51^ and 2nd mitochondrial dependency upon genetic loss of the KRAS oncogene,^41^ and connect them with a more generally described cancer cell state associated with therapy resistance, characterized by a dependence on lipid peroxidation defense pathways.^57,58^ Moreover, our work reinforces and extends the concept that PDAC progression and homeostasis rely on the active suppression of ferroptosis.^52^

Our study has several limitations. We deliberately chose a time- and resource-intensive autochthonous genetic mouse model to ensure a tumor-physiological microenvironment for preclinical translation. Achieving a further significant improvement in overall survival in the inherently fragile KPC model, beyond the already substantial effect of SHP2/MEK inhibition, is inherently challenging. Moreover, the development of more specific ferroptosis-inducing agents with optimized pharmacokinetic and pharmacodynamic properties for in vivo application remains an unmet need. Furthermore, we have not yet investigated crosstalk with the immune system and thus do not account for the dichotomous role that has been ascribed to ferroptotic cell death in promoting and suppressing the antitumor immune response.^59^ However, the mechanisms underlying heterogeneous treatment responses, independent of molecular subtype, remain poorly understood, and reliable predictive markers or biomarkers are still lacking. Finally, we cannot exclude the possibility that some of the effects observed are also related to dysregulated SHP2 activity, specifically in mitochondria. Allosteric inhibition of SHP2 might disrupt phosphorylation patterns in the respiratory chain, possibly enhancing mitochondrial activity and ROS production following combination therapy.^60–62^

In summary, our study demonstrated that interference with RAS signaling via combined SHP2/MEK inhibition provoked mitochondrial remodeling and oxidative stress both in vitro and in vivo, largely irrespective of the molecular PDAC subtype. These changes emerge early, already with short-term treatment, and persist into a therapy-resistant state that evolves with long-term inhibition. Notably, this phenotype is recapitulated by direct RAS-targeting compounds.

These metabolic adaptations increase the susceptibility of PDAC cells to ferroptotic cell death via lipid peroxidase inhibition, although contributions from other regulated cell death pathways cannot be excluded. Our findings provide a strong rationale for future translational studies exploring ferroptosis-inducing agents to overcome adaptive resistance to RAS-pathway inhibition.

## Materials and methods

### Cell culture summary

The human PDAC cell lines MIA PaCa II, PANC-1, and YAPC were cultured in RPMI 1640 medium supplemented with 10% FCS and 1% penicillin-streptomycin. Primary murine PDAC cells were derived from Kras^tm1Tyj^:*Kras*^*LSL-G12D/+*^ + Trp53^tm1Brn^:*Trp53*^*fl/fl*^ + Ptf1a^tm1(Cre)Hnak^:*Ptf1a*^*Cre-ex1/+*^ (KPC) compound mutant mouse tumors and were prepared as fragments for outgrowth culture. Additionally, primary murine PDAC cell lines (F2453 and F2683) from Kras^tm1Dsa^: *Kras*^*FSF-G12D/+*^*;* Trp53^tm1.1Dgk^: *Trp53*^*frt/frt*^*;* Ptf1a^tm1(flpo)Hcrd^: *Ptf1a*^*Flpo/+*^*;* Gt(ROSA)26Sor^tm3(CAG-Cre/ERT2)Dsa^*: R26*^*FSF-CreERT/+*^*;* Ptpn11^tm1Gsf^*: Ptpn11*^*fl/fl*^ (KPF-R26Cre-SHP2) compound mutant mice were isolated and subsequently subjected to *in vitro Ptpn11* deletion via 4-OH-tamoxifen. All the cells were maintained at 37 °C and 5% CO_2_ and were routinely tested for mycoplasma. To generate adaptive resistant cell lines, human and murine PDAC cells were cultured in RPMI 1640 with 10% FCS and 1% penicillin-streptomycin and treated continuously with fixed concentrations of SHP099, RMC-4550, trametinib, SHP099/trametinib, RMC-4550/trametinib, RMC-7977, or MRTX1133 as indicated for at least 8 weeks, with the media refreshed every 3 days.

### Patient-derived PDAC organoid (PDO) generation

Human PDAC tissue core biopsy samples were collected from surgical resection specimens. Tissue biopsies were minced, collagenase-treated, and ACK-lysed to isolate cells, which were then cultured as organoids in Matrigel domes with human feeding medium, following adapted protocols from the Tuveson Laboratory.^63,64^ All patients provided written informed consent prior to surgery. The study was performed in accordance with the Declaration of Helsinki and approved by the Ethics Committee of the University of Freiburg (No. 126/17 and No. 73/18). PDAC diagnosis was confirmed by routine pathology after resection of the pancreatic tumors.

### Plasmids and transfection

CRISPR-Cas9 constructs targeting the *PTPN11* gene were designed with pX458 vectors containing specific guide RNAs (gRNA 1 and gRNA 2). The oligonucleotide sequences used were as follows: gRNA 1 - forward: CACCGGAGGAACATGACATCGCGG, reverse: AAACCCGCGATGTCATGTTCCTCC; gRNA 2 - forward: CCACGAACATGACATCGCGGAGGTG, reverse: AAACCACCTCCGCGATGTCATGTTC. Each gRNA oligo pair was annealed and ligated into Bbs1-digested pX458 vectors. YAPC and PANC-1 *PTPN11* KOs were previously reported (ref. ^24^). MIA PaCa II cells were transfected with pX458-PTPN11-gRNA plasmids via the FuGENE® HD Transfection Reagent. Successfully transfected cells were sorted into singlets on the basis of GFP expression and grown in a 96-well plate. SHP2 KO clones, verified by Western blotting, were named by gRNA, e.g., MIA PaCa II KO2 for gRNA2.

### In vitro knockdown of GPX4

Human and murine PDAC cell lines were plated at a density of 26,000 cells/cm^2^ and allowed to adhere overnight. After 12–24 h, the cells were transfected with the respective siRNAs. The cells were treated with either a control siRNA (sc-37007) or a GPX4-targeting siRNA specific for human (sc-44465) or murine (sc-63302) cells. All siRNAs, the transfection reagent (sc-29528), and the transfection medium (sc-36868) were purchased from Santa Cruz Biotechnology. Transfections were performed according to the manufacturer’s instructions. For our experiments, 60 pmol of siRNA was used per 100 µL of siRNA transfection medium. Following transfection, the cells were cultured for 72 h.

### Mice

All experiments were conducted at the facilities of the Center for Experimental Models and Transgenic Services (CEMT) at the Medical Center University of Freiburg, Germany. The animals were maintained under hygienic, pathogen-free conditions, and all animal experiments and care were in accordance with local laws and the guidelines of institutional committees. The procedures were approved by the local authority (Regierungspräsidium Freiburg, Germany; approval number G/19-137). The authors complied with the “Animal Research: Reporting of In Vivo Experiments” (ARRIVE) guidelines. Genotyping was performed after weaning and after death.

### Drugs and antibodies

The drugs SHP099 (S8278), trametinib (S2673), RMC-4550 (S8718), and ML210 (S0788) were purchased from Selleckchem. RMC-7977 (HY-156498) and MRTX1133 (HY-134813) were purchased from MedChemExpress. The metabolism-related drugs oligomycin A (11342), rotenone (13995), antimycin A (19433), 2-deoxyglucose (14325), and 6-aminonicotinamide (10009315) were purchased from Cayman Chemical. Chloroquine (HY-17589A), PGC-1α-inhibitor (HY-101491), C75 (HY-12364), withaferin A (HY-N2065), and ferrostatin-1 (HY-100579) were purchased from MedChemExpress. Etomoxir (E1905), *tert*-butyl hydroperoxide (TBHP) (458139), and MitoTEMPO (SML0737) were obtained from Sigma‒Aldrich, and carbonyl cyanide-p-trifluoromethoxyphenylhydrazone (FCCP) (ab147482) was obtained from Abcam. Proteins were detected via western blotting with the following antibodies: pSTAT3 1:1000 (D3A7), STAT3 1:1000 (79D7), SHP2 1:1000 (D50F2), pAKTS473 1:1000 (D9E), AKT 1:1000 (C67E7), pERKT202/Y204 1:1000 (D13.14. E4), ERK 1:1000 (137F5), KRAS 1:1000 (E2M9G), KRASG12D 1:1000 (D8H7), KRT81 1:1000 (H00003887-M01J), ZEB1 1:1000 (D80D3), E-Cadherin 1:1000 (24E10), N-Cadherin 1:1000 (D4R1H), Vimentin 1:1000 (D21H3), GATA-6 1:1000 (D61E4), SLUG 1:1000 (C19G7), SNAIL 1:1000 (C15D3), GPX4 1:1000 (E5Y8K) and Actin 1:5000 (A2066). All the antibodies were purchased from Cell Signaling Technology except for KRT81 (Thermo Fisher Scientific) and Actin (Sigma-Aldrich).

### In vitro proliferation assay

For this cell proliferation assay, we cultured the following PDAC cells at the corresponding densities: murine KPC cells at 3.125 cells/cm^2^ surface area, human PANC-1 and MIA PaCa II cells at 5.800 cells/cm^2^ surface area, and human YAPC cells at 7.800 cells/cm^2^ surface area. The cells were plated overnight to allow attachment to the surface area. After 12–24 h, we added 15 μM SHP099, 15 μM RMC-4550, 10 nM trametinib, 10 nM RMC-7977 or a combination of an SHP2 inhibitor and trametinib. Additionally, we added metabolic inhibitors at the indicated concentrations. The experiment was terminated when the cells subjected to the respective targeted therapy reached a cell density of approximately 90% (the cells were treated for approximately 6–14 days). At the end of the experiment, the cells were fixed with 3.8% formaldehyde and stained with a solution of crystal violet (20% Thanol+0.2% crystal violet from Sigma‒Aldrich). Fixed and stained cells were imaged with a ChemiDoc^TM^ MP Imaging System from Bio-Rad, and the mean gray value was determined via ImageJ version 1.53a.

### In vitro proliferation of human PDAC organoids

A total of 1000 cells were mixed with 5 μl of Matrigel, and one dome was placed in each well of a 96-well plate. Organoids were cultured in human feeding medium for 72 h. On the following day, treatment with 15 μM SHP099, 25 nM trametinib, 10 nM RMC-7977, or a combination of SHP099 and trametinib was initiated. Additionally, the organoids were treated with metabolism-associated inhibitors at the indicated concentrations. After 5 days of treatment, organoid proliferation was quantified via a BrdU colorimetric immunoassay from Roche (Cat. No. 11 647 229 001) or the CellTiter-Glo® 2.0 Cell Viability Assay from Promega (G9241). Owing to the involvement of metabolic pathways targeted by the inhibitors, as shown in Supplementary Fig. 14e, this assay was performed via BrdU incorporation. In contrast, the results shown in Fig. 5 and Supplementary Fig. 14f were obtained via CellTiter-Glo.

### Determination of ML210 IC50 values under pathway inhibition and rescue conditions

To evaluate ferroptosis sensitivity under RAS/MAPK pathway inhibition, cells were treated with increasing concentrations of the GPX4 inhibitor ML210 (0.25, 1, 2, 5, 10, and 20 µM; final DMSO ≤ 0.1%) in the presence of either DMSO, RMC-7977 (10 nM), or vertical MAPK pathway blockade (combination: 10 nM trametinib + 15 µM RMC-4550). Where indicated, the cells were cotreated with the ferroptosis inhibitor ferrostatin-1 (2 µM) or the mitochondrial ROS scavenger MitoTEMPO (20 µM). Cell viability was assessed by crystal violet staining. For each treatment group (DMSO, RMC-7977, or Combo), the assay was terminated once the corresponding cells *without* ML210 reached approximately 90% confluence. The absorbance was measured and normalized to that of the respective treatment control (without ML210). IC_50_ values for ML210 were calculated via nonlinear regression with a variable slope (four-parameter logistic model) in GraphPad Prism (v9.2.0).

### Flow cytometry

Human and murine PDAC cells were seeded and allowed to adhere overnight. For intracellular ROS measurements, the cells were treated with 15 μM SHP099, 10 nM trametinib, or both for 72 h. Intracellular ROS levels were assessed via a BD LSRFortessa flow cytometer with a 20 nm bandpass at 712 nm. To induce ROS, the cells were treated with 100 μM tert-butyl hydroperoxide (TBHP) 30 min before harvesting. TBHP-treated cells were used as a positive control. To determine the mitochondrial mass, the cells were stained with MitoTrackerTM Deep Red FM following similar steps. For lipid peroxidation analysis, the cells were treated with 15 μM RMC-4550, 10 nM trametinib, 10 nM RMC-7977, 10 μM MRTX1133, or a combination of an SHP2 inhibitor and trametinib for 48 h. Sixteen hours prior to analysis, the cells were treated with ML210 at the indicated concentrations or with 2 μM ferrostatin-1 as an antioxidant control. Lipid peroxidation was detected via BODIPY™ 581/591 C11 (Thermo Fisher Scientific) staining and analyzed on a BD LSRFortessa flow cytometer with a 20 nm bandpass filter centered at 510 nm.

### Immunoblotting

For immunoblotting, the cells were lysed with RIPA buffer, and the protein concentrations were determined. The lysates were denatured, separated via SDS‒PAGE, and transferred to nitrocellulose membranes. After blocking, the membranes were incubated with primary and secondary HRP-conjugated antibodies and then visualized with a ChemiDoc system (Bio-Rad).

### Oxygen and glucose consumption

To measure the OCR and ECAR of PDAC cells, 3×10⁴ PANC-1 cells were plated in a poly-D-lysine precoated XF96 microplate and allowed to attach overnight. The cells were treated for 24 h with 15 μM SHP099, 10 nM trametinib, or their combination. On the day of measurement, the medium was replaced with DMEM containing glucose (25 mM) and glutamine (2 mM) without sodium bicarbonate (pH 7.4), and the mixture was incubated for 30–60 min at 37 °C in a CO₂-free incubator. Mitochondrial stress (Seahorse 101706-100) and glycolysis stress tests (Seahorse 103020-100) were performed according to the manufacturer’s instructions, and the OCR and ECAR were normalized to the cell number via the CyQUANT™ (Thermo Fisher Scientific) assay.

### Histology and immunohistochemistry

Tissue samples were fixed in 4% paraformaldehyde, dehydrated, and embedded in paraffin. Sections (4 μm) were deparaffinized and subjected to antigen retrieval with 10 mM citrate buffer (pH 6.0), followed by blocking with 3% hydrogen peroxide. Core staining was performed with Hemalaun. Immunohistochemistry was performed with an anti-MDA antibody (1:300 Abcam, Ab27642), and the signal intensity was analyzed via QuPath (v0.2.2). To interpret the signal intensity of tumor cells, a histoscore was calculated that consists of the sum of (1 × % weak staining) + (2 × % moderate staining) + (3 × % strong staining).

### Transcriptomics

To analyze transcriptomic profiles, cells were treated with 15 μM SHP099, 10 nM trametinib, or their combination. After 48 h, the cells were lysed in Qiagen RLT Plus buffer and purified via the RNeasy® Plus Mini Kit. In addition to parental cell lines, resistant derivatives and bulk RNA-seq samples derived from KPC tumor tissue were included in the analysis. Sequencing was conducted at the German Cancer Research Center (DKFZ) via an Illumina NovaSeq™ 6000. The data were converted to FASTQ files, quality trimmed, and aligned to reference genomes (GRCh38 for human, GRCm39 for mouse) with the STAR aligner (v2.7.10a).^65,66^ Differential expression analysis was performed via limma, and GSEA was carried out via the clusterProfiler R package^67^ and MSigDB collections.^68–70^ PDAC subtypes were classified on the basis of five key studies (Moffitt, Bailey, Collisson, Puleo, Chan-Seng-Yue). To facilitate integrative analyses, we defined two overarching categories—basal union and classical union—by consolidating subtype signatures corresponding to the basal-like and classical phenotypes, respectively. Subtype-specific scores were computed for each (untreated) sample via single-sample gene set enrichment analysis (ssGSEA), implemented through the clusterProfiler package. Prior to ssGSEA, gene expression values were scaled across the entire cohort. Human PDAC signatures were mapped to their murine orthologs via the homologene R package.^71^ PDAC subtype classification was subsequently performed following the same analytical framework applied to the human cohort. Cell type deconvolution was performed with CIBERSORTx to estimate the cell type composition of 20 mouse PDAC samples via a custom 13-cell type reference matrix.^72^ The analysis scripts and subtype signatures used in this study are publicly available in the GitHub repository.^73^

### Proteomics

To analyze proteomic profiles, cells were treated with 15 μM SHP099, 10 nM trametinib, or their combination. After 72 h, the cells were lysed in 50 mM triethylammonium bicarbonate + 5% SDS buffer supplemented with Halt^TM^ Protease Inhibitor Cocktail (Thermo Fisher Scientific). Proteomic samples were then prepared, TMT labeled, and fractionated following the methods of Alatibi et al.^74^ For LC‒MS/MS, 800 ng of peptide was analyzed on a Q Exactive Plus mass spectrometer connected to an EASY-nLCTM 1000 UHPLC system. The column configuration included an Acclaim™ PepMap™ C18 column and a 200 cm µPac GEN1 analytical column, with peptide separation achieved via a 120-min gradient from 5 to 100% buffer B (0.1% formic acid in 80% acetonitrile). Peptides were analyzed in data-dependent acquisition mode, with survey scans at 70,000 resolution and targeted fragmentation of the top 10 precursor ions at 17,500 resolution. The data were processed via MaxQuant v1.6.14.0 with the Human-EBI database (downloaded January 9, 2020).^74^

### Preparation of samples for metabolomics analysis

For quantitative metabolite profiling, SHP2-expressing cells were treated with 15 μM SHP099, 10 nM trametinib, or their combination for 12, 48, or 72 h. In addition, SHP2 KO cells were analyzed in parallel under baseline conditions. Following treatment or harvesting, the cell pellets were lysed in DPBS supplemented with 1% protease inhibitor cocktail (Sigma, P8340-5ML). Whole-cell lysates and cell culture media were then aliquoted for polar metabolite analysis, acylcarnitine profiling, and protein quantification and stored at −80 °C. The levels of the sulfur-containing metabolites creatinine, SAM, and SAH were quantified via previously described methods.^75,76^ Lactate, TCA and glycolysis intermediates, organic acids, folates, amino acids, and neurotransmitters were profiled following established protocols.^77^ Amino acid calibration curves were generated from a standardized mixture, while other metabolites were calibrated from in-house stock solutions. Accuracy was confirmed by monitoring specific metabolites in external quality controls and validated plasma samples, with data collected on a Sciex 6500+ ESI-tripleQ MS/MS coupled to a Nexera ultra-performance liquid chromatograph. Acylcarnitines were extracted from tissue homogenates, categorized by chain length, and analyzed via a Sciex 5500+ MS/MS. Quantification was performed with Analyst® 1.7.2 software.

### Preparation of tumor interstitial fluid for metabolomics

To isolate TIF, tumors were excised from treated KPC animals as quickly as possible, washed with PBS, and then completely blotted dry. The tumors were subsequently centrifuged at 200 × *g* for 10 min at 4 °C using an EASY strainer™ (20 µm, Greiner BIO-ONE). The isolated interstitial fluid was stored at −80 °C, and the levels of sulfur-containing metabolites were quantified as previously described.^75,76^ Lactate, TCA, and glycolysis intermediates, and other organic acids, folates, and amino acids were determined as described in previous work.^77,78^ Folates, TCA intermediates, and amino acids (including urea cycle intermediates) were calibrated with a standardized amino acid mixture. For extraction, 20 µL of fluid was treated with DTT, incubated, and processed with 0.1% formic acid in methanol. Calibration was validated with external quality controls, and quantification was carried out via Analyst® 1.7.2 software on an LC‒MS/MS system.

### Single-cell sequencing

Treated KPC tumors were harvested, minced, digested with collagenase II (C6885, Sigma, 5 mg/mL in RPMI) for 20 min at 37 °C, and filtered through a 100 µm and 70 µm cell strainer. The cells were stained with anti-CD45 AF700 (BioLegend, 103128) for 20 min on ice and sorted via the BC Cytoflex SRT. For single-cell mRNA barcode labeling, the GEXSCOPE® Single-cell RNA Library Kit Cell V2 Kit from Singleron Biotechnologies GmbH was used, and cDNA was sent for library preparation and sequencing via an Illumina PE 150. The alignment and quantification of single-cell RNA-seq data were performed via CeleScope (Singleron Biotechnologies, version 1.14.0), and the data were aligned to the mouse genome “Mus_musculus_ensembl_92” (Ensembl). Count matrices were analyzed with Scanpy (version 1.9.1).^79^ Quality control excluded cells with fewer than 500 genes, more than 25,000 UMI counts, or more than 10% mitochondrial counts. Gene expression was log-normalized and scaled to 10,000.^80^ PCA was performed on the top 5000 variable genes, followed by Harmony integration to correct for batch effects.^81^ Leiden clustering (resolution 0.5) was used, and UMAP was employed for visualization.^82,83^ Differentially expressed genes (DEGs) were identified via the Wilcoxon rank-sum test, with adjusted *p* values ≤ 0.05 and log fold changes ≥ 1. GO enrichment analysis was performed with ClusterProfiler in R^67,84,85^ (version 4.2.2) and GSEAPY for curated gene sets^86^ in the scRNA-seq data, which were visualized in UMAP.

### Embedding and preparation of pancreatic tissue for electron microscopy

Pancreatic tissue was embedded in Tissue-Tek^®^ O.C.T. compound (Sakura Finetek), snap-frozen, and stored at −80 °C until further use. On Day 1, the pancreatic tissue was fixed in 4% paraformaldehyde and 2.5% glutaraldehyde, followed by washing and osmication. After dehydration, the tissue was incubated in uranyl acetate overnight. On Day 2, the tissue was subjected to ethanol treatment, propylene oxide immersion, and embedding in Durcupan. On Day 3, the tissue was cured, sectioned, and stained. Semithin (300 μm) sections were stained with toluidine blue, while ultrathin (58 nm) sections were contrasted with lead citrate. The samples were sectioned on a Leica Ultracut UC6 and analyzed with a Zeiss LEO 906E transmission electron microscope.

### Statistical analysis

Statistical analysis was conducted via GraphPad PRISM 9.2.0 software. For experiments analyzing only one variable across multiple conditions, ordinary one-way ANOVA with correction for multiple comparisons was employed. Groups or samples were considered significantly different when *P* < 0.05. Detailed statistical information for each experiment is provided in the corresponding supplementary information. Large-dimensional data from omics experiments were examined with R Studio using the indicated R packages.^87^

### Grammatical and stylistic editing

To improve clarity and precision, grammatical and stylistic revisions were made with the assistance of an AI-based language model (ChatGPT, OpenAI), which was used exclusively for language-related support. No content-related changes or data analyses were performed via this tool.

## Supplementary information

Supplementary Material (PDF)

Metabolomics Data

## Data Availability

Transcriptomic and single-cell RNA-sequencing datasets generated in this study are publicly available. Bulk RNA-seq data can be accessed at [https://zenodo.org/records/16631280], and single-cell RNA-seq data can be accessed at [https://zenodo.org/records/16636353].
