# Supplementary material for: Vertical RAS pathway inhibition in pancreatic cancer drives therapeutically exploitable mitochondrial alterations
Source: Signal Transduct Target Ther. 2026 Jan 16;11:33. doi: 10.1038/s41392-025-02563-7 (PMC12811380; doi:10.1038/s41392-025-02563-7)
Supplement: Supplementary file 1 — Supplementary Material (PDF) [file 41392_2025_2563_MOESM1_ESM.pdf]

Supplementary Materials for

Vertical RAS pathway inhibition in pancreatic cancer drives therapeutically exploitable mitochondrial alterations

Philipp Hafner, Steffen J. Keller, Xun Chen, Asma Alrawashdeh, Huda Jumaa, Friederike I. Nollmann, Solène Besson, Judith Kemming, Oliver Gorka, Tonmoy Das, Bismark Appiah, Ariane Lehmann, Mujia Li, Petya Apostolova, Bertram Bengsch, Robert Zeiser, Stefan Tholen, Oliver Schilling, Olaf Groß, Andreas Vlachos, Uwe A. Wittel, Dominik von Elverfeldt, Wilfried Reichardt, Melanie Boerries, Geoffroy Andrieux, Guus J. Heynen, Stefan Fichtner-Feigl, Luciana Hannibal, Dietrich A. Ruess

Correspondence to: **dietrich.ruess@uniklinik-freiburg.de**

This PDF file includes:

- Figures. S1 to S15

Supplementary Figure 1

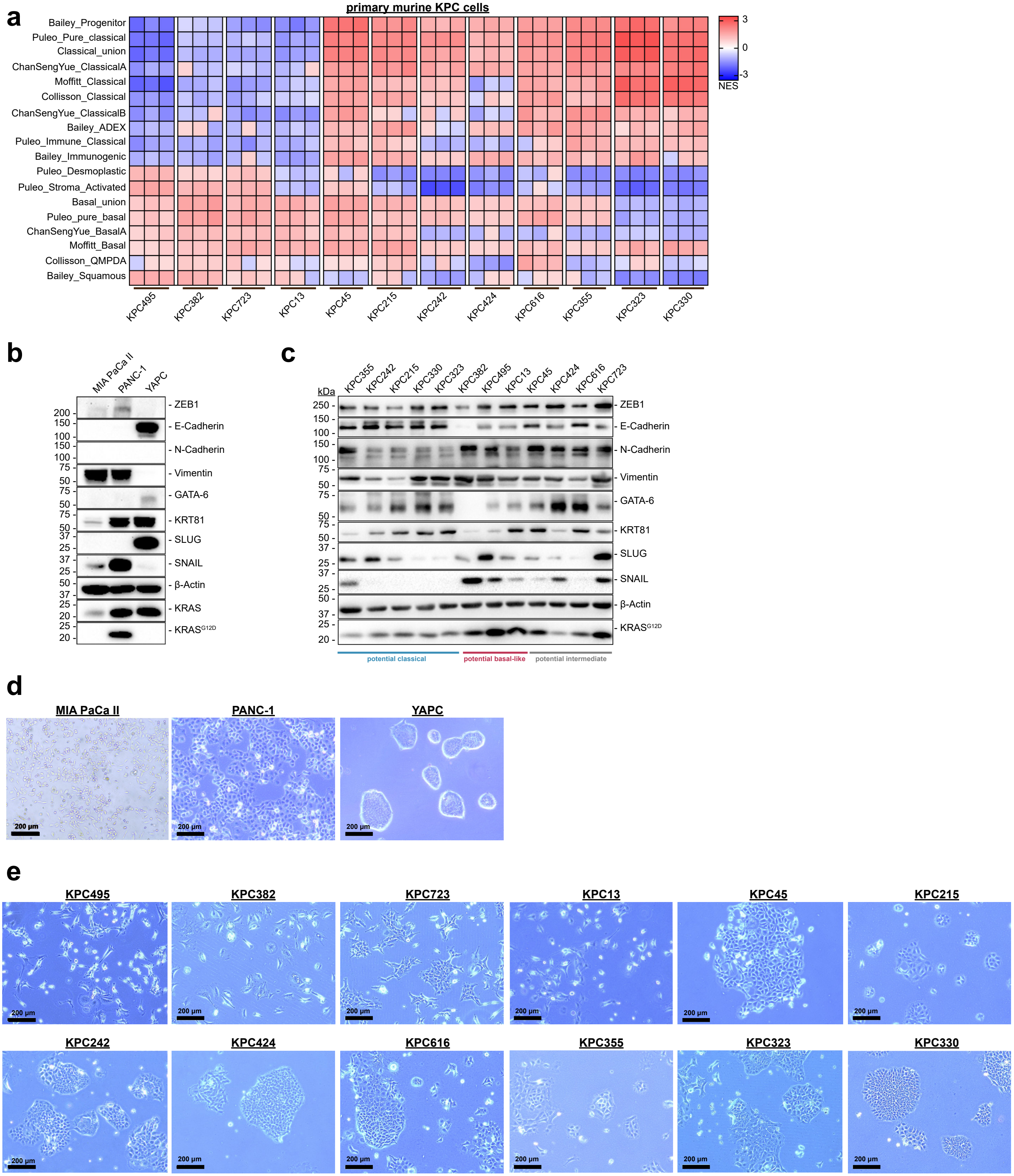

Supplementary Figure 1: Classification of murine KPC and human PDAC cell lines by molecular subtype. (**a**) Transcriptomic analysis of human PDAC cell lines and murine KPC cells classifying them into basal-like or classical molecular subtypes based on ssGSEA. Color code represents the Normalized Enrichment Score (NES) of each PDAC subtype signature. (**b**) Western Blot analysis of human PDAC cell lines for subtype related proteins. β-actin served as housekeeping protein. (**c**) Western Blot analysis of murine KPC cell lines for subtype related proteins. β-actin served as housekeeping protein. The protein lysates were generated between passage 5-10. (**d** and **e**) Bright field micrographs showing morphology of human PDAC and murine KPC cell lines, respectively.

Supplementary Figure 2

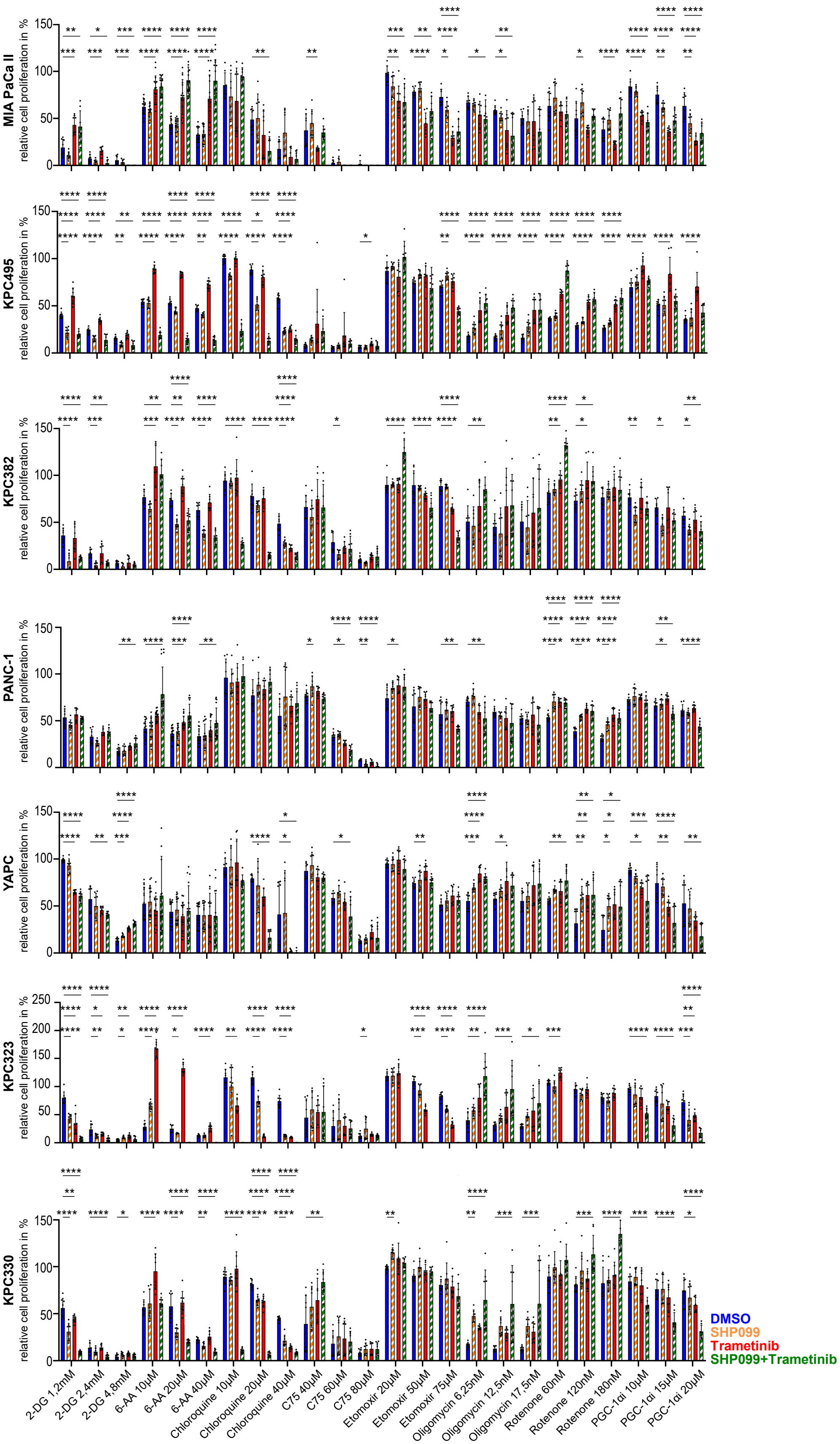

Supplementary Figure 2: Screening of metabolic inhibitors in the context of SHP2/MEK blockade. Proliferation assay of human PDAC cell lines and murine KPC cell lines. Cells were incubated for 5-8 days and treated with either DMSO, SHP099 (15 µM), trametinib (10 nM), or combination of SHP099 and trametinib. Additionally, the cells were treated with metabolic inhibitors at the depicted concentrations. To investigate the influence of the metabolic inhibitors on viability on the background of SHP2 +/- MEK inhibitor treatment, the values given were calculated in relation to those resulting from SHP2 +/- MEK inhibitor treatment without metabolic inhibition. Statistical significance was determined via one-way ANOVA with comparisons made only against corresponding DMSO controls. * $P < 0.05$, ** $P < 0.01$, *** $P < 0.001$, **** $P < 0.0001$.

Supplementary Figure 3

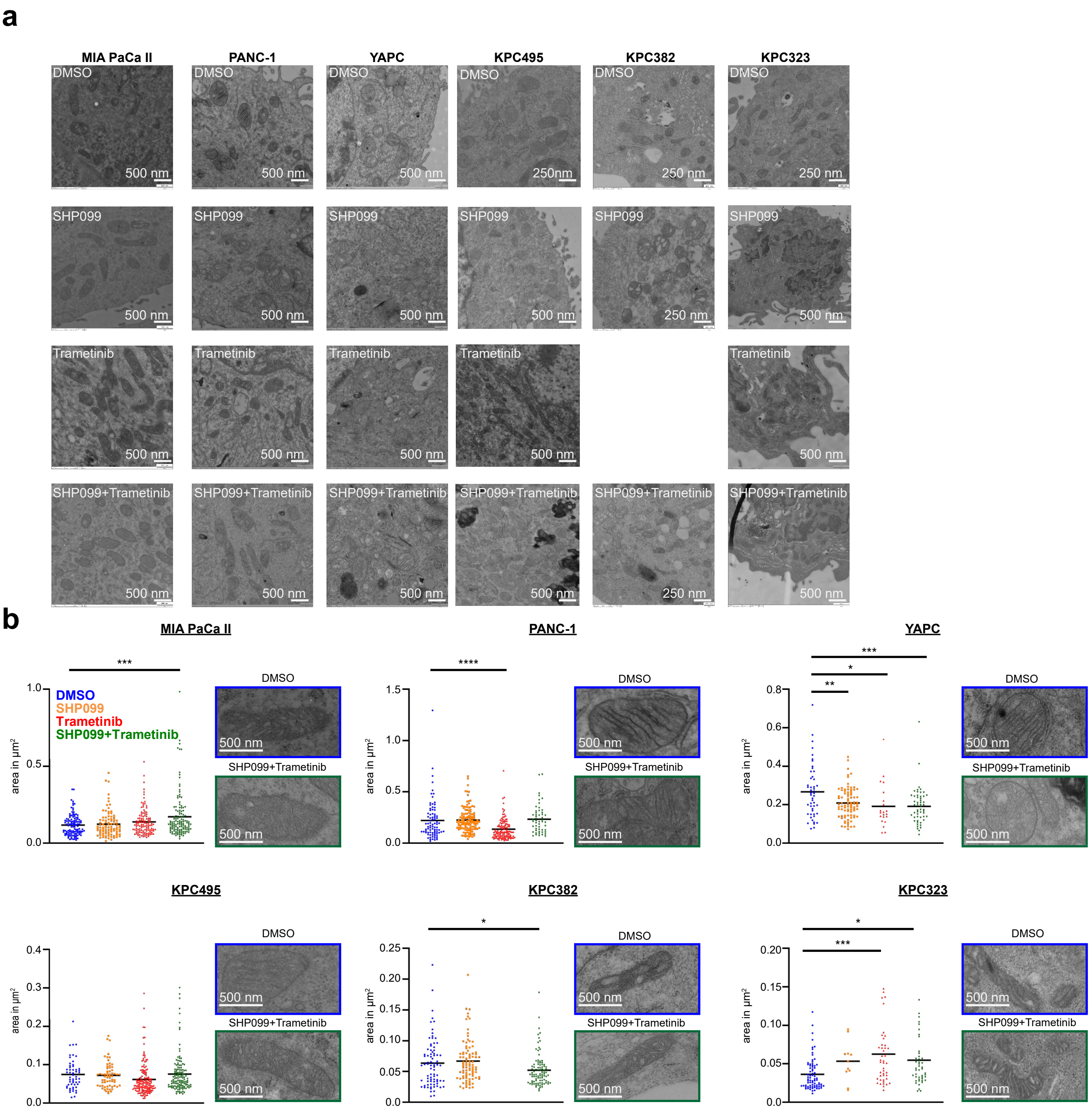

Supplementary Figure 3: Dual SHP2/MEK inhibition induces changes in mitochondrial size and shape. (**a**) Electron microscopy images of human PDAC cell lines and murine KPC cell lines. Cells were treated for 72 hours with either DMSO, SHP099 (15 μM), trametinib (10 nM) or the combination of SHP099 and trametinib. (**b**) Mitochondrial diameter of human PDAC cell lines and murine KPC cell lines based on electron microscopy. Statistical significance was assessed using one-way ANOVA in panel b with comparisons made against corresponding DMSO controls. * $P < 0.05$, ** $P < 0.01$, *** $P < 0.001$, **** $P < 0.0001$.

Supplementary Figure 4

a

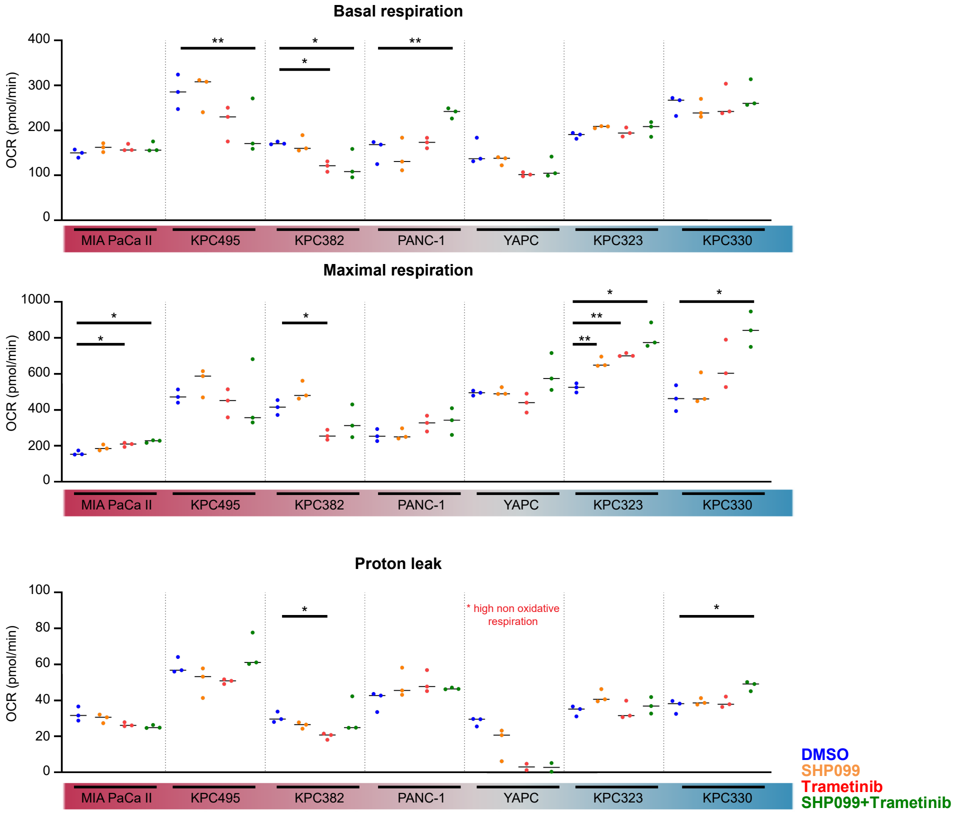

b

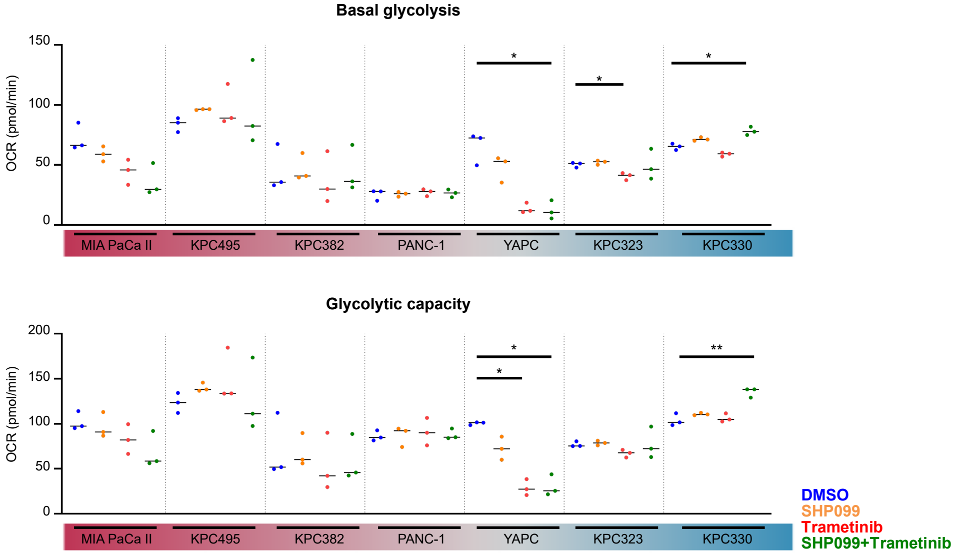

Supplementary Figure 4: Dual SHP2/MEK inhibition alters cellular respiration and glycolysis. (**a**+**b**) Before measurement, human PDAC cell lines and murine KPC cell lines were treated for 72 hours with either DMSO, SHP099 (15 μM), trametinib (10 nM) or the combination of SHP099 and trametinib. (**a**) Respiration analysis based on oxygen consumption rate. Mitochondrial function was assessed using the Agilent Seahorse XF Cell Mito Stress Test. (**b**) Glycolysis analysis based on extracellular acidification rate. Glycolytic function was measured using the Agilent Seahorse XF Glycolysis Stress Test. Data are shown as the mean of 3 independent experiments, with each biological replicate consisting of 6 technical replicates. Statistical significance was determined via one-way ANOVA in panels a and b, with comparisons made against corresponding DMSO controls. * $P < 0.05$, ** $P < 0.01$, *** $P < 0.001$, **** $P < 0.0001$.

Supplementary Figure 5

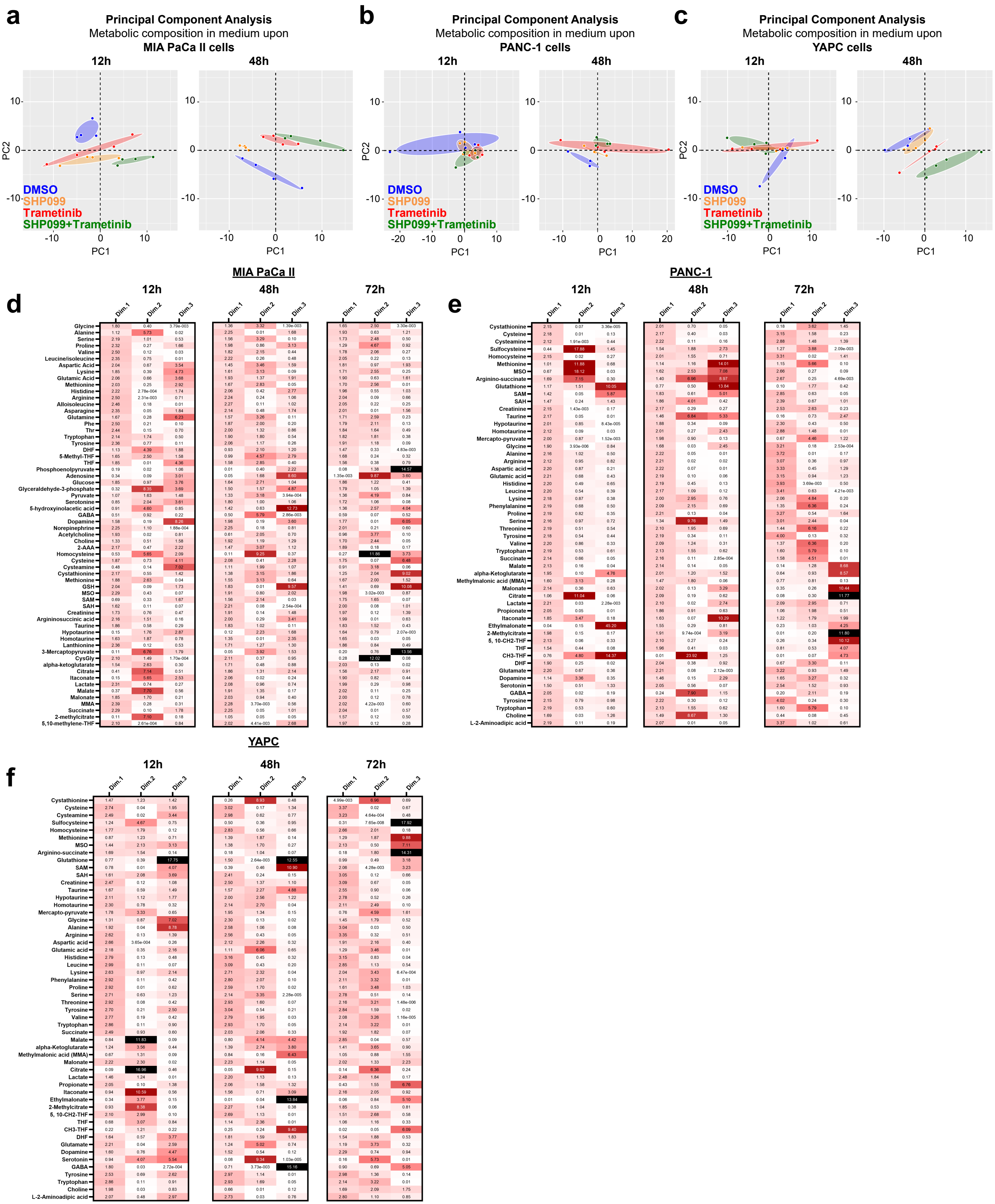

Supplementary Figure 5: SHP2/MEK blockade modulates the *in vitro* metabolome. (**a-c**) Principal component analysis of extracellular metabolites determined via LC-MS/MS metabolomics. Human PDAC cell lines were treated for 12 hours and 48 hours with either DMSO, SHP099 (15 µM), trametinib (10 nM) or the combination of SHP099 and trametinib. Four independent samples are shown per treatment. Number of included metabolites for PCA: 62 for MIA PaCa II cells, 54 for PANC-1 and YAPC cells. (**d-f**) PCA contribution factors for human PDAC cell lines at 12, 48, and 72 hours of treatment. The PCA contribution factors display the loadings of each variable on the principal components from the PCA. The contribution factors indicate how much each variable influences the principal components. Lower-dimensional principal components have a greater impact on the data, as they account for more of the variance.

Supplementary Figure 6

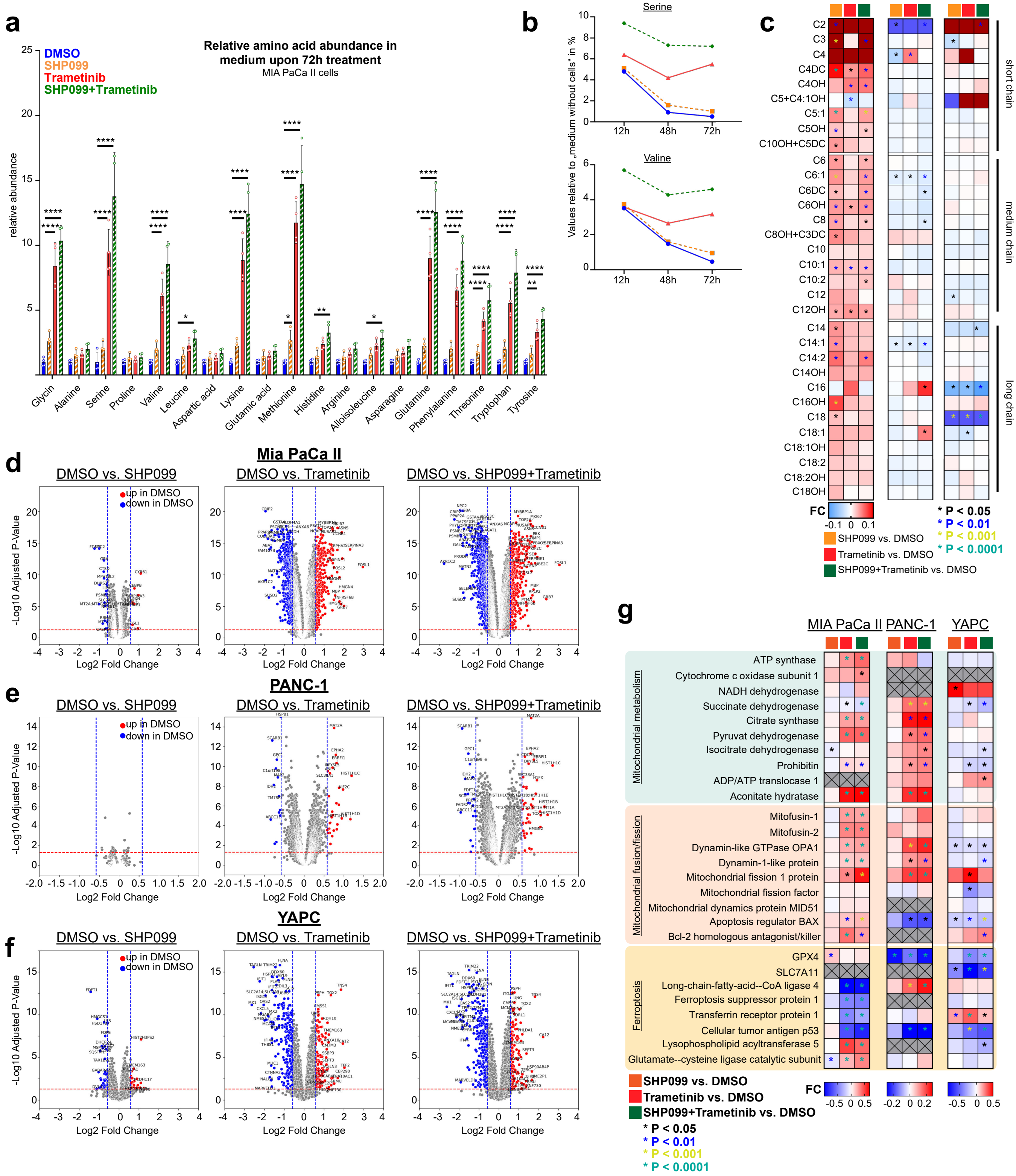

Supplementary Figure 6: Metabolomic and proteomic analysis reveals metabolic adaptions with SHP2 +/- MEK inhibition *in vitro*. (**a-c**) Metabolomics. (**d-g**) Proteomics. (**a**) Extracellular amino acid abundance in MIA PaCa II cells after 72 hours of treatment with SHP099 (15 μM), trametinib (10 nM), or both. (**b**) Two representative extracellular amino acids in MIA PaCa II relative to medium control. (**c**) Acylcarnitine levels in PDAC cell lines after 48-hour treatments. Values > 0.1 in dark red, < −0.1 in dark blue for clarity. (**d-f**) Proteomic profiling of human PDAC cell lines demonstrates altered protein abundance following 72 hours of treatment with SHP099 (15 μM), trametinib (10 nM), or their combination, compared to control (DMSO). Displayed are proteins with Log2 fold changes < -0.58 or > 0.58, and adjusted P-values < 0.05. (**g**) Protein composition analysis indicates changes in mitochondrial metabolism, mitochondrial dynamics (fusion/fission), and ferroptotic response after 72 hours of treatment with SHP099, trametinib, or their combination. Statistical significance for panels d-f was determined using limma with a t test. Statistical significance was determined via one-way ANOVA in panels a and g with comparisons made against corresponding DMSO controls. *P < 0.05, **P < 0.01, ***P < 0.001, ****P < 0.0001. For panel c statistical analysis was performed with R[1] and the R package limma[2]. * P < 0.05, ** P < 0.01, *** P < 0.001, **** P < 0.0001.

1 Ritchie, M. E. *et al.* limma powers differential expression analyses for RNA-sequencing and microarray studies. *Nucleic Acids Res.* 43, e47-e47, (2015).

2 Team, R. C. R: A language and environment for statistical computing [Computer software]. (2021).

Supplementary Figure 7

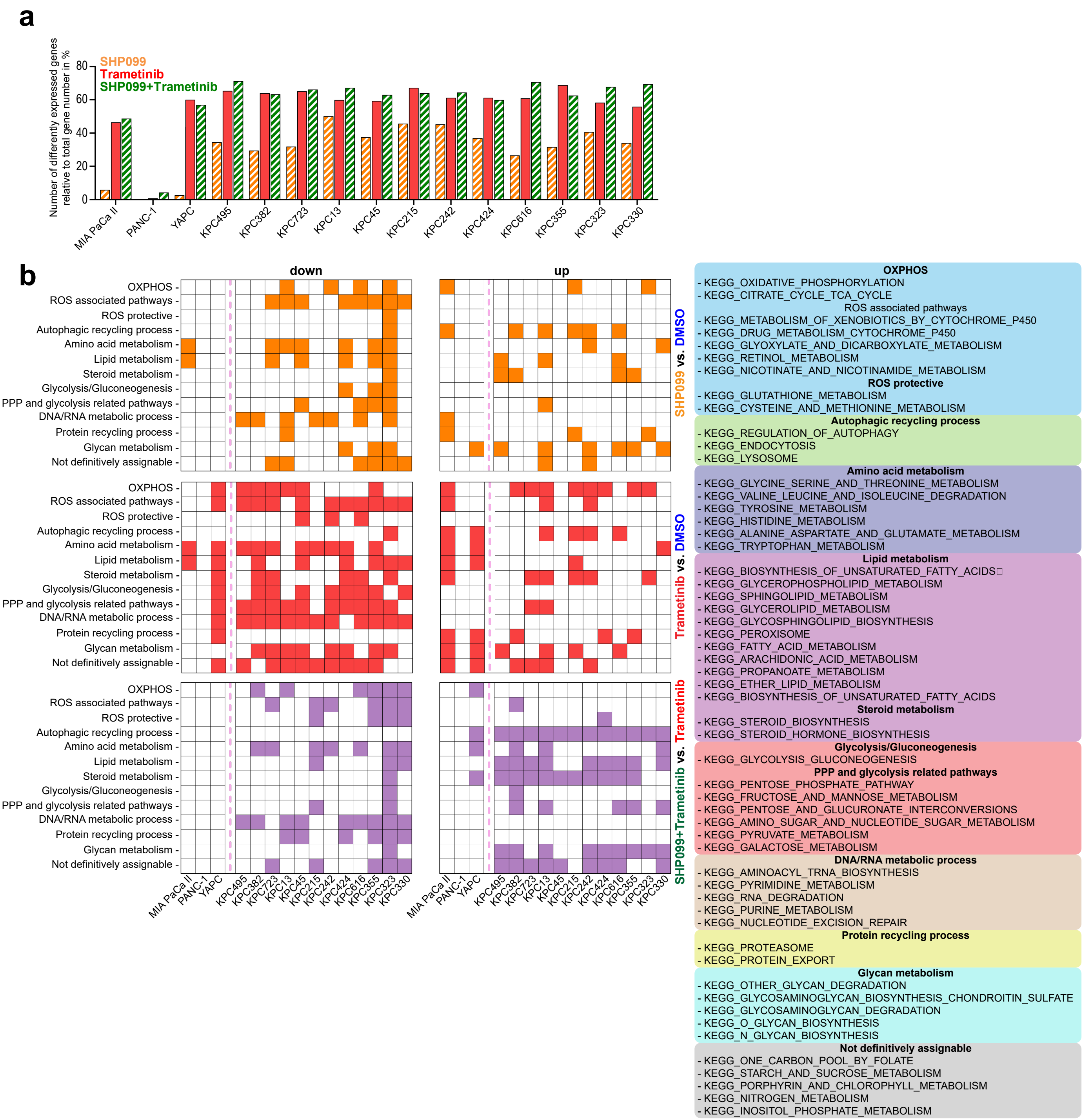

Supplementary Figure 7: Transcriptomic analysis highlights mitochondrial and broader metabolic adaptations under MAPK pathway inhibition. (**a**) Number of differentially expressed genes (DEGs) in human PDAC and murine KPC cell lines treated for 48 hours with SHP099 (15 μM), trametinib (10 nM), or both; DEGs with adj. $P < 0.01$ are shown. (**b**) KEGG-based gene set enrichment analysis (GSEA) of human and murine cell lines. Each colored square represents a significant upregulation or downregulation of at least one gene set relative to DMSO, as depicted from the right. "Not definitively assignable" indicating unclear metabolic classification. Gene sets with adj. $P < 0.25$ are indicated.

Supplementary Figure 8

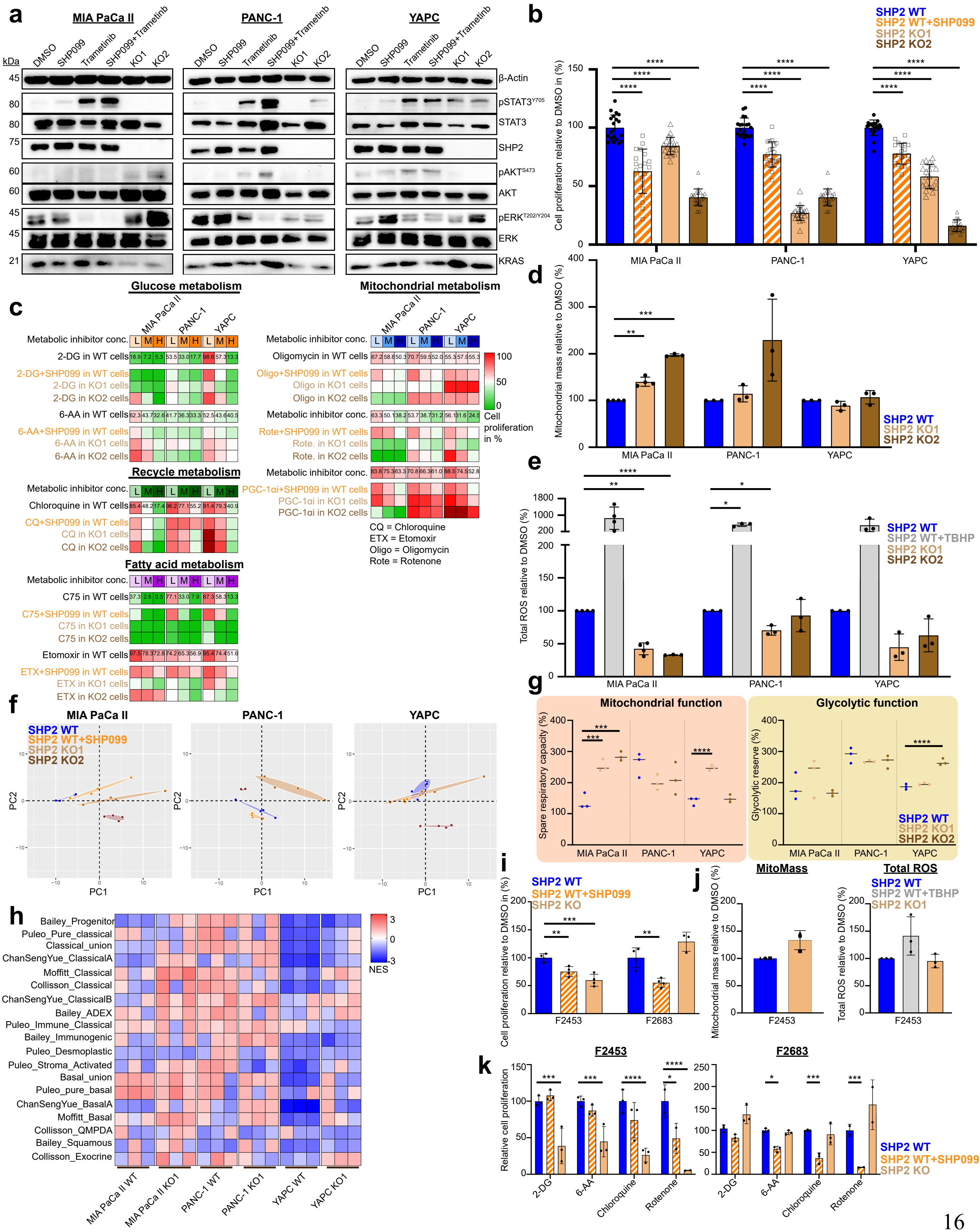

Supplementary Figure 8: Genetic deletion of PTPN11 (SHP2) promotes subtype plasticity and distinct metabolic adaptation. (**a**) Western Blot analysis of human PDAC cell lines with and without genetic SHP2 KO. (**b**) Cell proliferation of SHP2 expressing cells and SHP2 KO cells for a period of 10 days. Error bars represent the SD of 20 wells from one experiment. The sensitivity to SHP2 treatment varied in this experiment compared to that depicted in Figure 1b. Here, the initial cell count was substantially lower, rendering them more sensitive to SHP099 in terms of proliferation compared to cells in Figure 1b. (**c**) Relative cell proliferation of SHP2-KO cells in the presence of metabolic inhibitors (L: low, M: medium, H: high concentration). Metabolic inhibitor concentrations correspond to those used in Supplementary Fig. 2. Data for SHP2-expressing cells were extracted from the experiments shown in Fig. 1d-g. Each colored area of the heatmap represents the mean of 8 wells from two independent experiments. (**d**) Flow cytometry determination of mitochondrial mass in human PDAC cell lines SHP2-proficient vs. SHP2-deficient, relative to cell size. Each data point represents 1 of 3-4 independent experiments. (**e**) Flow cytometry determination of intracellular total reactive oxygen species in human cells, SHP2-proficient vs. SHP2-deficient. Each data point represents 1 of 2-4 independent experiments. (**f**) PCA of metabolomics data from SHP2-proficient vs. SHP2-deficient human PDAC cell lines, including 72 h treatment of the WT lines with SHP099 in comparison. 4 independent samples were analysed per condition. Number of metabolites included for PCA analysis for MIA PaCa II cells: 62; for PANC-1 and YAPC cells: 54. (**g**) Spare respiratory capacity and glycolytic reserve of human SHP2 WT and SHP2 KO cells, based on ECAR and OCR values. Mitochondrial function was assessed using the Agilent Seahorse XF Cell Mito Stress Test and glycolytic function was measured using the Agilent Seahorse XF Glycolysis Stress Test. Data are shown as the mean of three independent experiments. (**h**) Transcriptomic analysis of human PDAC cell lines and murine KPC cell lines classifying them into basal-like or classical molecular subtypes based on ssGSEA. Color code represents the Normalized Enrichment Score (NES) of each PDAC subtype signature. (**i**) Cell proliferation of primary murine PDAC cells. Each data point represents one independent experiment. (**j**) Flow cytometry determination of mitochondrial mass and total ROS in murine SHP2-expressing and SHP2 KO cells relative to cell size. Each data point represents 1 of 3 independent experiments. (**k**) Relative proliferation of SHP2 expressing cells treated with 15 µM SHP099 or SHP2 deleted cells in presence of metabolic inhibition. Values are relative to the respective DMSO treated control group. Statistical significance was determined via one-way ANOVA in panels b, d, e, g, i, j and k, with comparisons made only against corresponding DMSO controls. * $P < 0.05$, ** $P < 0.01$, *** $P < 0.001$, **** $P < 0.0001$.

Supplementary Figure 9

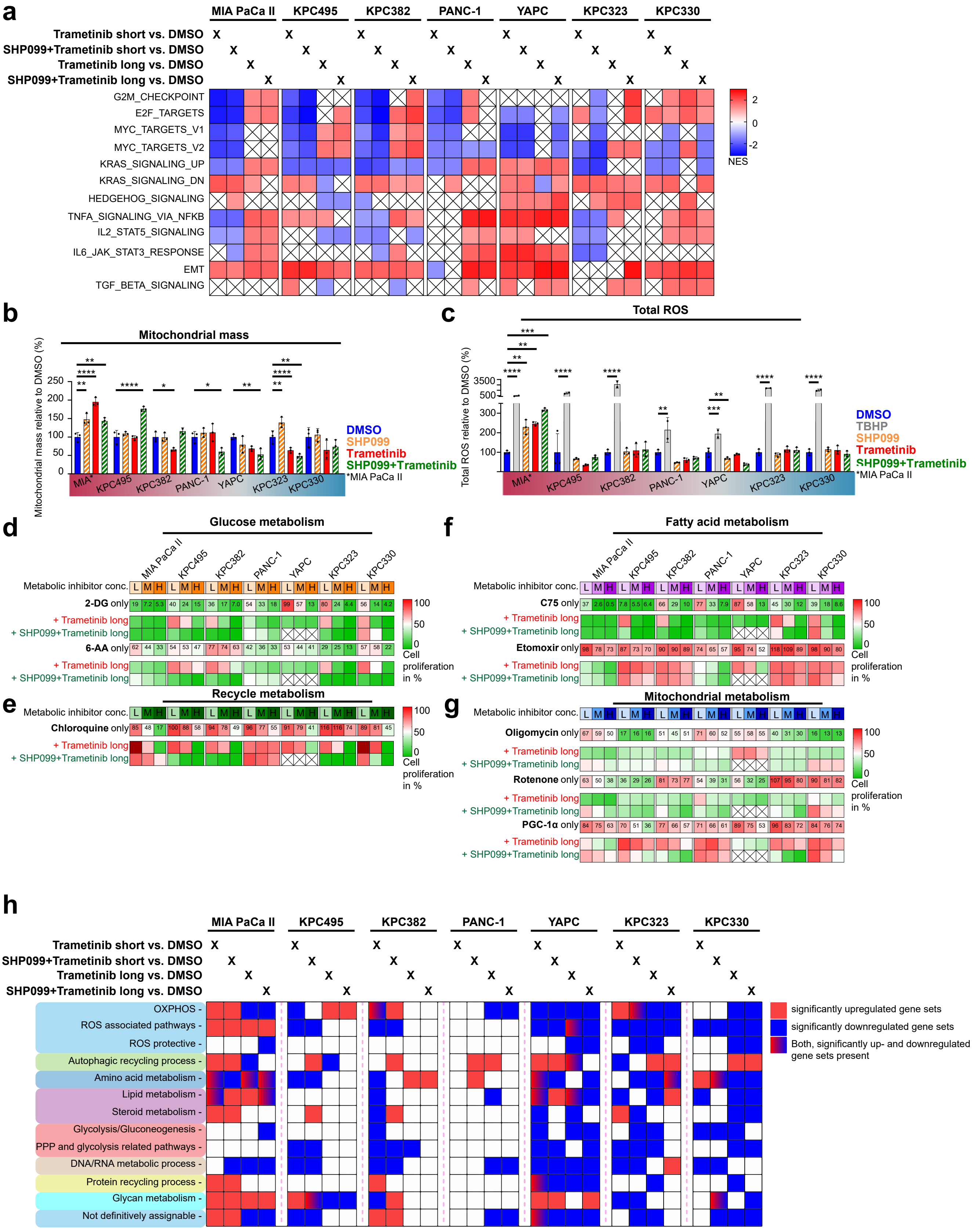

Supplementary Figure 9: Adaptive resistance and persistent mitochondrial remodeling following long-term MEK and SHP2/MEK blockade *in vitro*. (**a**) Transcriptomic analysis of human and murine PDAC cell lines treated for 48 hours (short) or at least 8 weeks (long) with either trametinib (10 nM) or a combination of SHP099 (15 µM) and trametinib (10 nM). Gene set enrichment analysis (GSEA) was performed using HALLMARK gene sets, with adjusted P-values < 0.25. The data represent treated cells relative to control cells. (**b**) Flow cytometric analysis of mitochondrial mass in continuously treated human PDAC cell lines, normalized to cell size. Each data point represents one of two to three independent experiments. (**c**) Flow cytometric analysis of intracellular total reactive oxygen species (ROS) in continuously treated human PDAC cell lines, normalized to cell size. Each data point represents one of two to three independent experiments. (**d-g**) Relative proliferation in the presence of metabolic inhibitors (L: low, M: medium, H: high concentration) in resistant human PDAC cell lines. Each colored area of the heatmap represents the mean of 8 wells from two independent experiments. Data for cells treated with only metabolic inhibitors were derived from Fig. 1 d-g. Metabolic inhibitor concentrations are similar to those from supplementary Fig. 2. YAPC cells continuously treated with the combination therapy exhibited very slow proliferation and therefore could not be used in this approach. (**h**) Gene set enrichment analysis of human and murine PDAC cell lines treated for 48 hours (short) or at least 8 weeks (long) with either trametinib (10 nM) or a combination of SHP099 (15 µM) and trametinib (10 nM) based on the KEGG gene set collection. Up- or downregulation is indicated by the corresponding squares, with gene sets having adjusted P-values < 0.25 highlighted. All white squares for these cell lines or treatment conditions are not statistically significant and are therefore excluded from the analysis. Statistical significance in panels b and c was determined via one-way ANOVA, with comparisons made only against corresponding DMSO controls. * P < 0.05, ** P < 0.01, *** P < 0.001, **** P < 0.0001.

Supplementary Figure 10

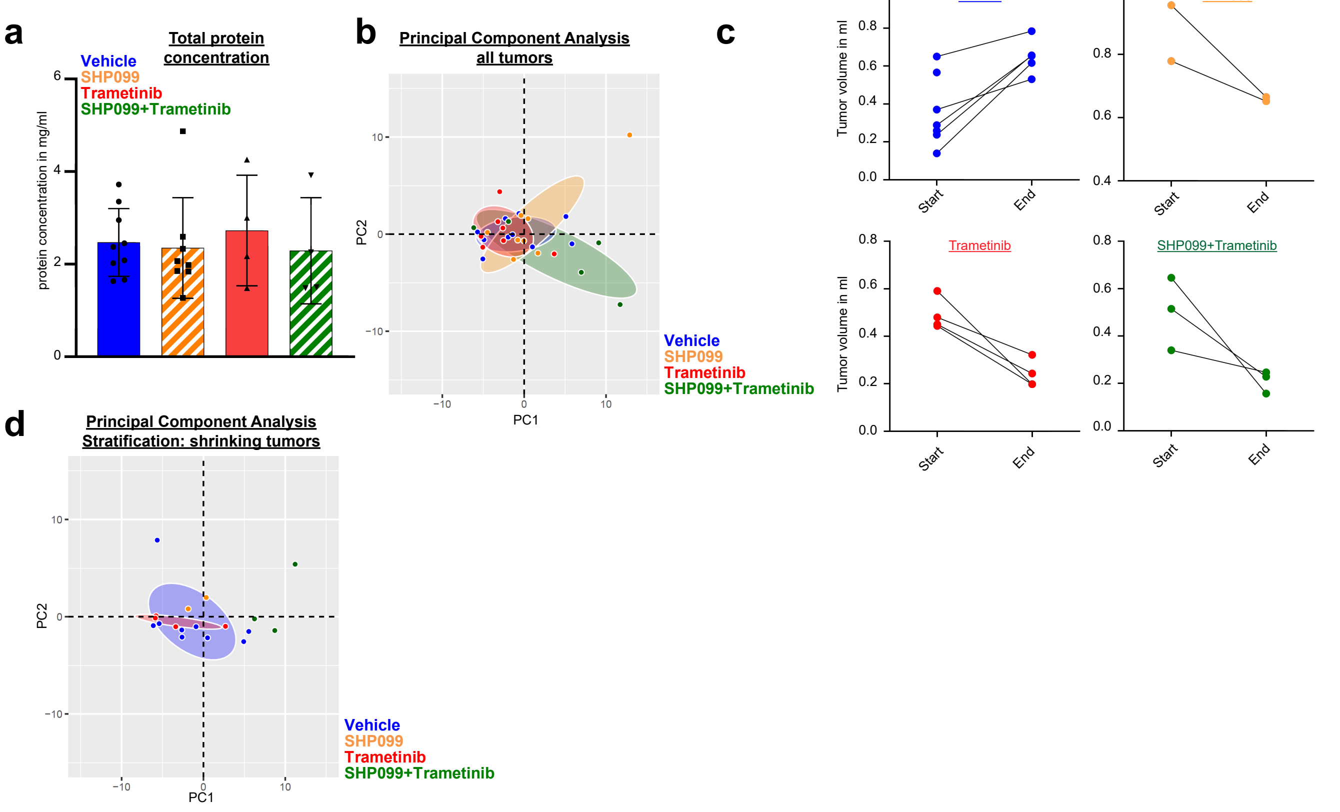

Supplementary Figure 10: Metabolite profiling of tumor interstitial fluid (TIF) in KPC tumors *in vivo*. (**a**) TIF protein concentration in different therapy groups. (**b**) PCA of 63 metabolites from TIFs in KPC tumors under therapy. (**c**) PDAC tumor development of shrinking tumors using magnetic resonance imaging. (**d**) PCA, as in b, of tumors stratified by shrinkage behavior.

Supplementary Figure 11

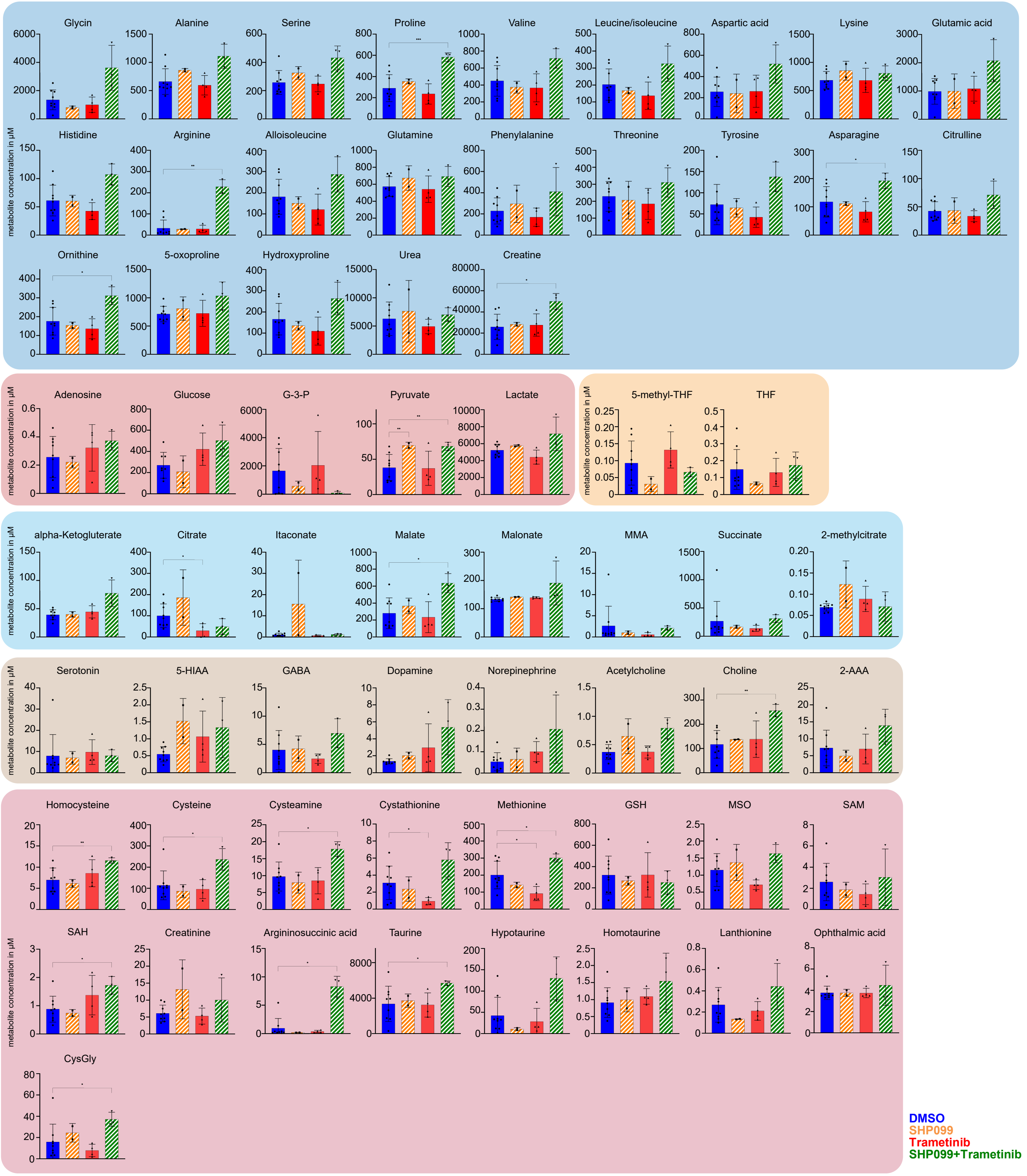

Supplementary Figure 11: SHP2/MEK inhibition modulates tumor interstitial fluid (TIF) metabolites *in vivo*. TIFs from freshly sacrificed animals were utilized for measurement via LC-MS/MS. Animals were treated either with vehicle, SHP099 (75mg/kg), trametinib (1mg/kg) or the combination of SHP099 and trametinib. Statistical significance was determined via one-way ANOVA, with comparisons made only against corresponding DMSO controls. * $P < 0.05$, ** $P < 0.01$, *** $P < 0.001$, **** $P < 0.0001$.

Supplementary Figure 12

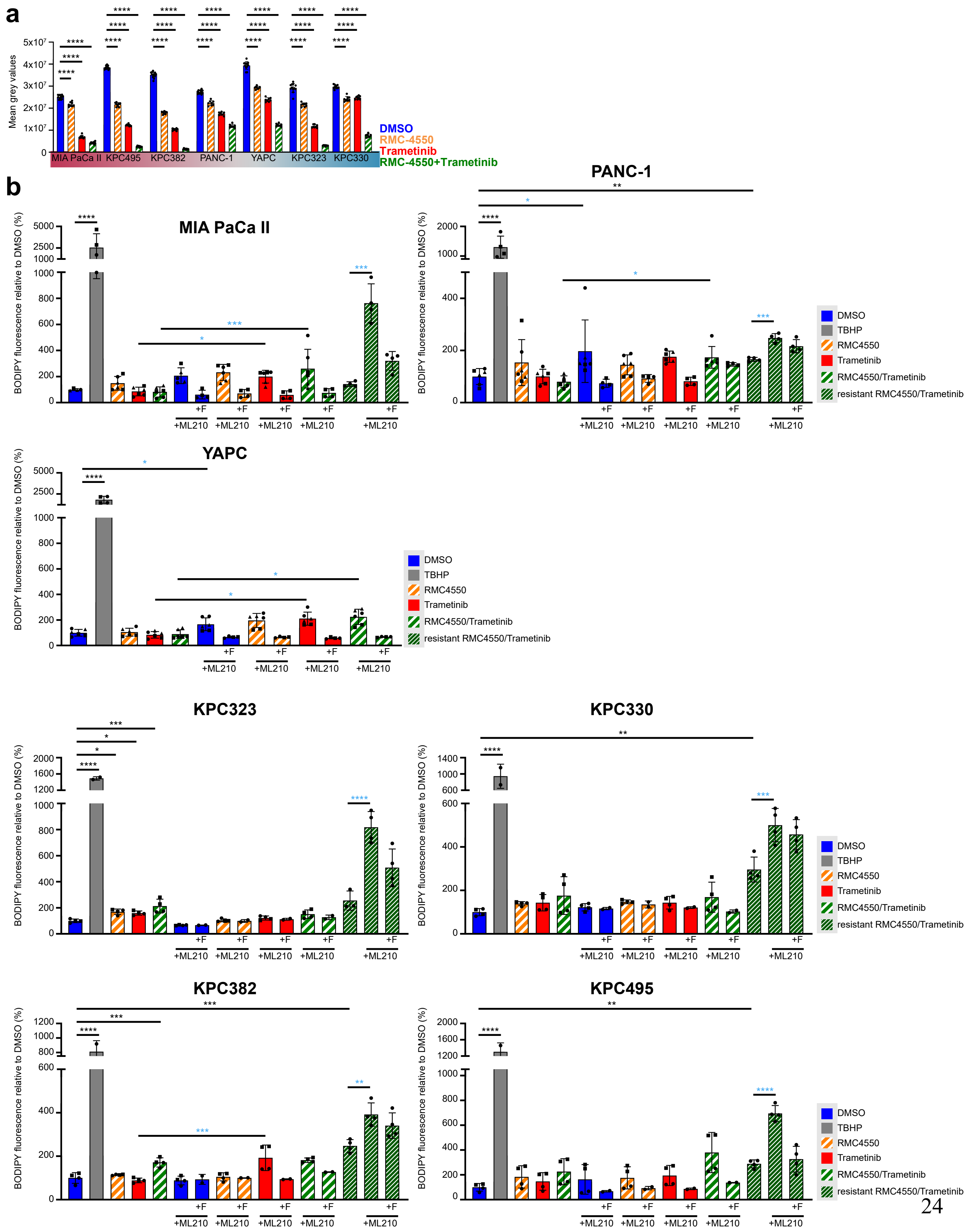

Supplementary Figure 12: Analysis of lipid peroxidation levels measured by C11-BODIPY fluorescence *in vitro*. (**a**) Cell proliferation of human PDAC cell lines and murine KPC cell lines treated with DMSO, RMC-4550 (15 μM), trametinib (10 nM), or both. Error bars show SD of 8 wells from one experiment. (**b**) Human PDAC and murine KPC cell lines were treated for 48 hours with DMSO, RMC-4550 (15 μM), trametinib (10 nM), or their combination. Resistant cells were generated by continuous exposure to the same treatment for at least 8 weeks. 16 hours prior to measurement, cells were treated with ML210 at the following concentrations: PANC-1 (0.1 μM), MIA PaCa II (0.25 μM), KPC495 (5 μM), YAPC & KPC330 (10 μM), and KPC323 (5 μM). As a negative control, cells were treated with the antioxidant Ferrostatin-1 (2 μM) for 16 hours. For the positive control, cells were exposed to TBHP for 15-30 minutes. The depicted values are relative to DMSO control cells. Data represent two to three independent experiments with two technical replicates each; resistant cells represent one experiment with four technical replicates. Statistical significance was determined using one-way ANOVA. Black asterisks indicate statistically significant differences compared to the corresponding DMSO control, while blue asterisks indicate comparisons between targeted treatments with and without ML210. $^{*}P < 0.05$, $^{**}P < 0.01$, $^{***}P < 0.001$, $^{****}P < 0.0001$.

Supplementary Figure 13

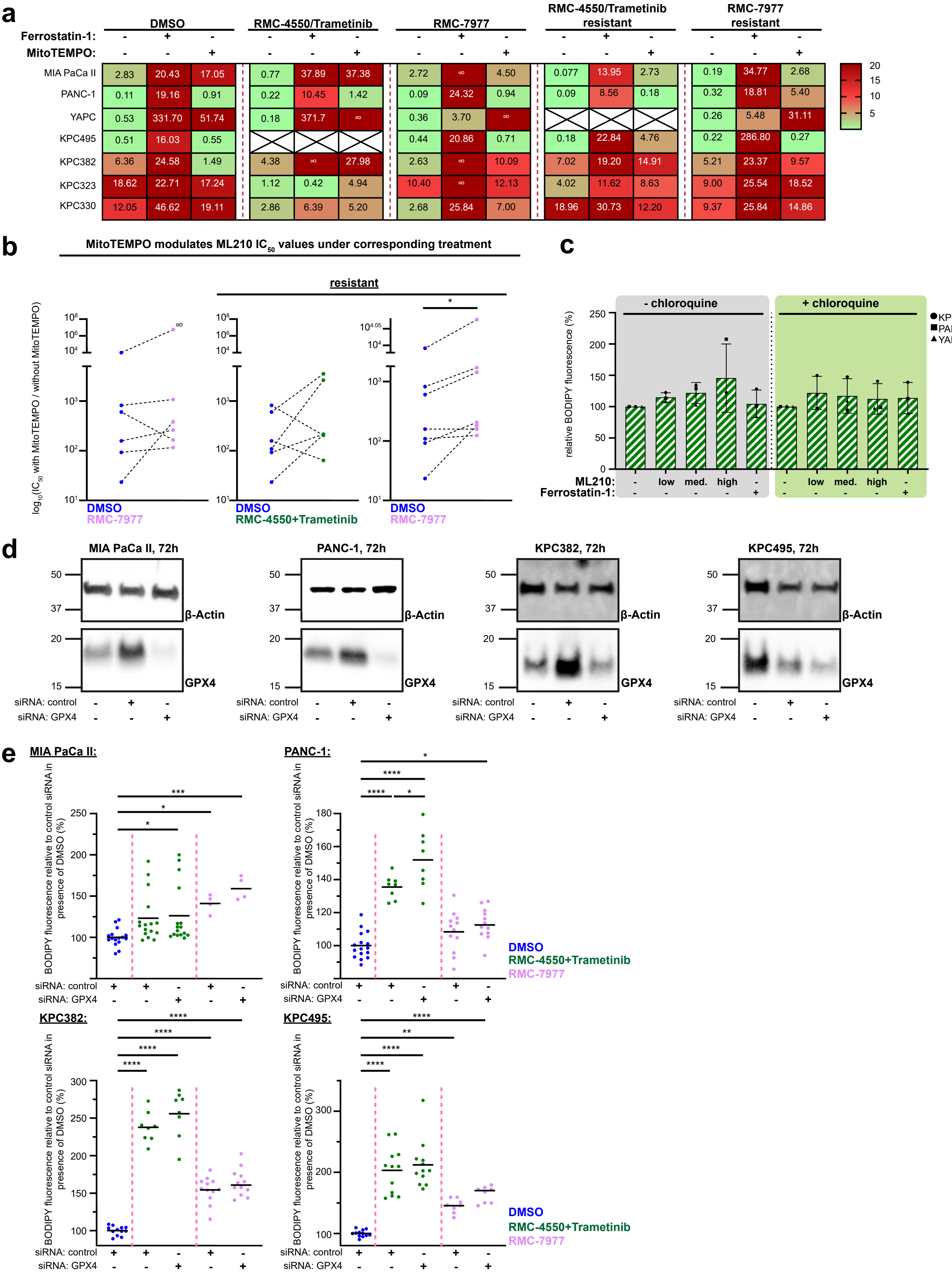

Supplementary Figure 13: Vulnerability to ferroptosis induction via GPX4 inhibition as a consequence of SHP2/MEK or pan-RAS inhibition *in vitro*. (**a**) IC_{50} values of the GPX4 inhibitor ML210 in the presence of either DMSO, the pan-RAS inhibitor RMC-7977 (10 nM), or vertical MAPK pathway blockade (trametinib 10 nM + RMC-4550 15 µM) in naïve and treatment resistant cell lines. Cells were additionally treated with Ferrostatin-1 or MitoTEMPO as indicated. (**b**) ΔIC_{50} values for human and murine PDAC cell lines treated with the indicated treatment conditions are shown, calculated as IC_{50} in the presence of MitoTEMPO relative to IC_{50} without. Values are plotted on a logarithmic scale. (**c**) Detection of lipid peroxidation via C11-BODIPY fluorescence. Effect of adding chloroquine (10 µM) to SHP2/MEK inhibition (48h). ML210 concentrations were adapted to individual cell line sensitivity. Low/Med/High concentrations - KPC382: 50/75/100 µM; PANC-1: 0.1/0.25/1 µM; YAPC: 10/15/20 µM. Experiments were conducted in 3 independent PDAC cell lines, each measured in two technical replicates (indicated as mean value) per condition. (**d**) Western blot analysis of GPX4 siRNA knockdown in PDAC cell lines after 72 hours. β-actin served as a housekeeping protein. (**e**) Detection of lipid peroxidation in human and murine PDAC cell lines after 72 hours of siRNA knockdown and ± RMC-4550 (15 µM) + trametinib (10 nM) or RMC-7977 (10 nM) treatment. Each dot represents an independent measurement. Statistical significance in panel b was determined by paired t test. Statistical significance in panel c and e was determined by one-way ANOVA: * $P < 0.05$, ** $P < 0.01$, *** $P < 0.001$, **** $P < 0.0001$.

Supplementary Figure 14

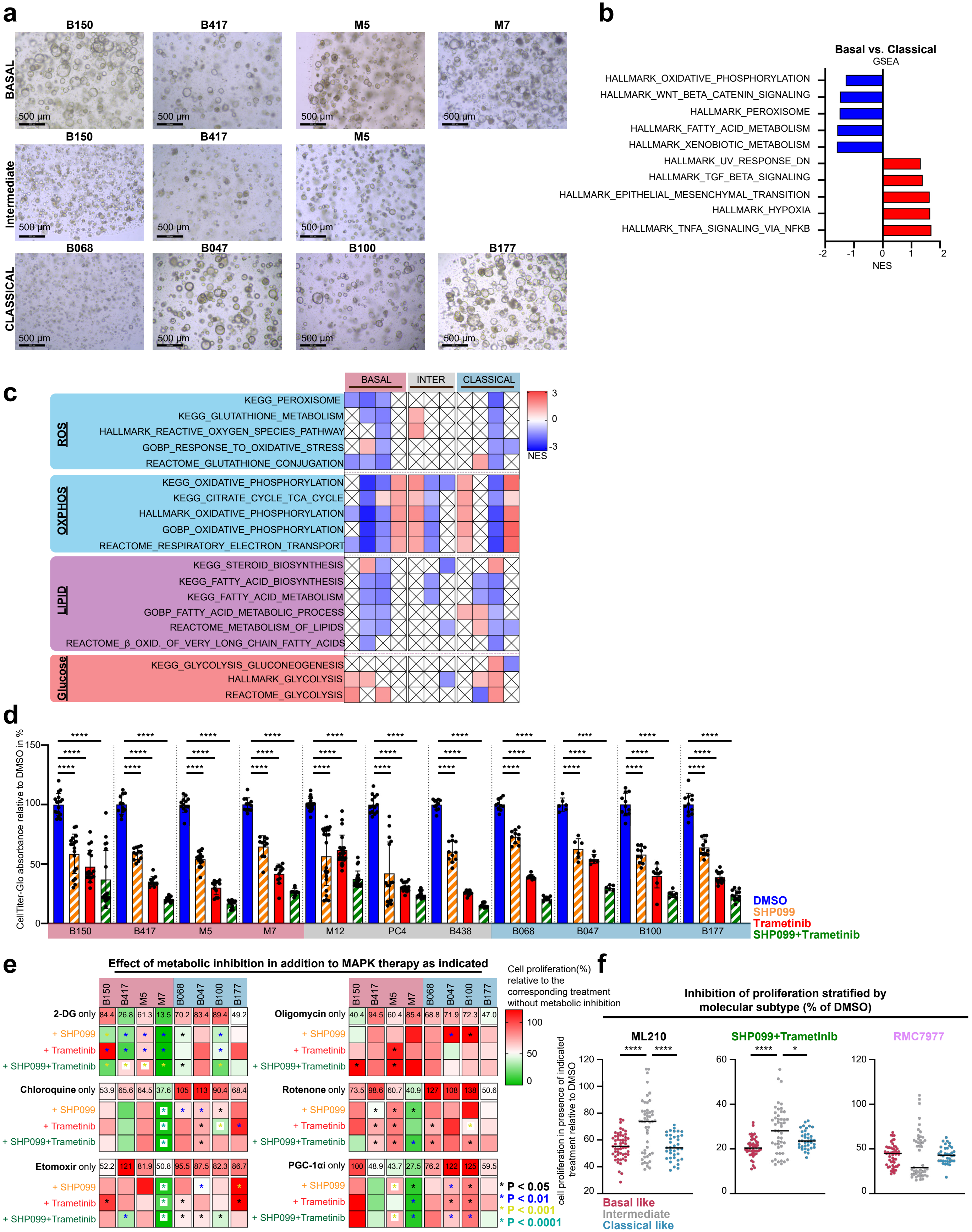

Supplementary Figure 14: Mitochondrial adaptations in patient-derived PDAC organoids in response to SHP2/MEK inhibition. (**a**) Morphology of human PDAC-derived organoids. (**b**) GSEA comparing basal-like and classical subtypes using HALLMARK gene sets. (**c**) KEGG-based GSEA of patient derived organoids. Color code represents the Normalized Enrichment Score (NES) of each PDAC subtype signature. Gene sets with adj. P < 0.25 are indicated. (**d**) Organoid proliferation in the presence of DMSO, SHP099 (15 µM), trametinib (25 nM), or their combination, with two to three biological replicates (three to five samples each). (**e**) Relative cell proliferation with or without MAPK inhibition plus metabolic inhibition. The data illustrate the effect of the metabolic inhibitor (e.g., 2-DG) on top of each MAPK pathway inhibition. Data are normalized to the corresponding MAPK pathway inhibition without metabolic inhibitor (e.g., SHP099+2-DG vs. SHP099). Heatmap represents the mean of 4 samples. Metabolic inhibitor concentrations: 2-DG (2 mM), chloroquine (20 µM), etomoxir (50 µM), oligomycin (30 µM), PGC-1αi (10 µM), rotenone (20 nM). (**f**) Proliferation-inhibitory effects of the indicated condition, displayed according to molecular subtype. Statistical significance (d, e, f) was assessed via one-way ANOVA. In panels d and e, effects were analyzed solely in comparison to DMSO controls: * P < 0.05, ** P < 0.01, *** P < 0.001, **** P < 0.0001.

Supplementary Figure 15

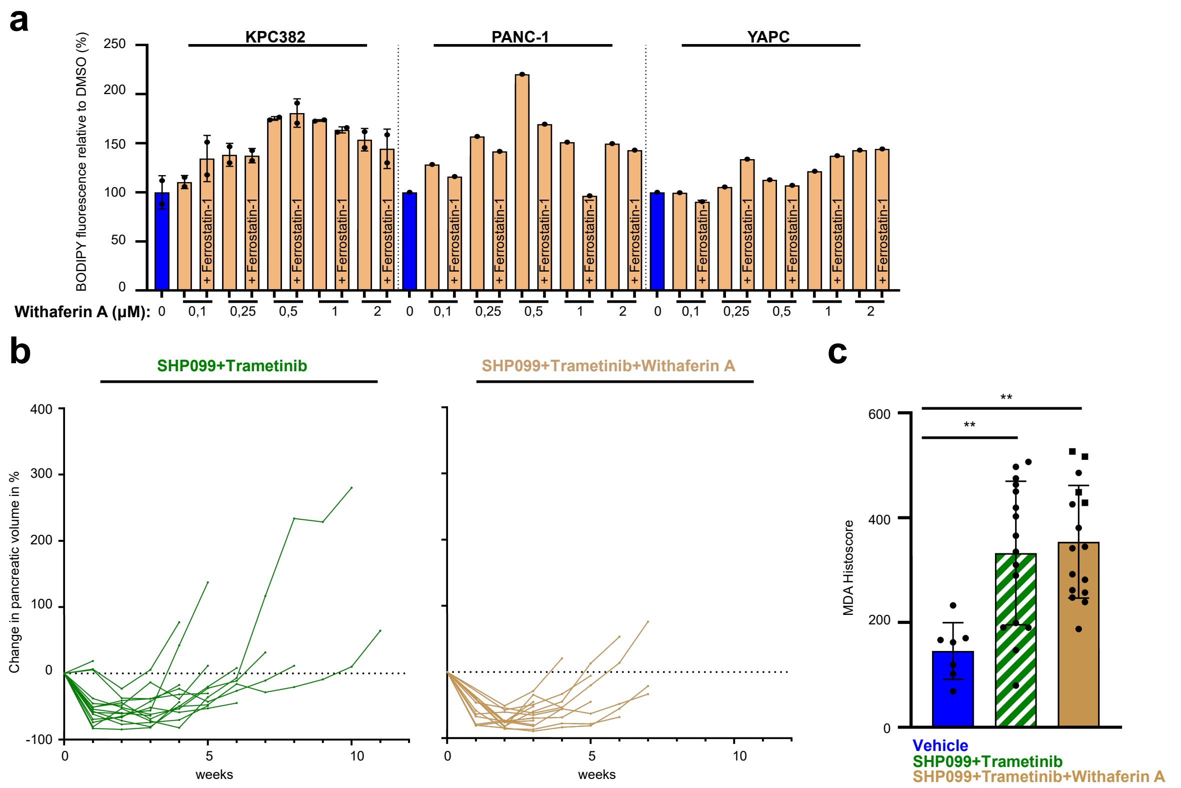

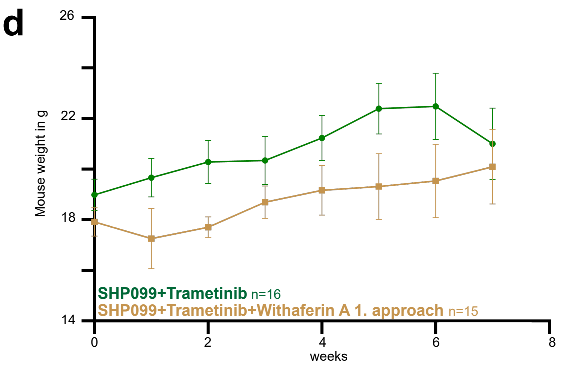

e

Frequency of termination criteria

	Vehicle n=15	SHP099 n=18	Trametinib n=20	SHP099 + Trametinib n=33	SHP099 + Trametinib + Withaferin A n=15
Ataxia (%):	6,7	16,7	15,0	6,0	26,7
Jaundice (%):	6,7	11,1	15,0	6,0	-
Abdominal enlargement (%):	33,3	22,2	30,0	24,2	26,7
Weight loss (%):	13,3	-	10,0	15,2	13,3
Asthenia/found dead in cage (%):	40,0	50,0	30,0	48,6	33,3

Supplementary Figure 15: Effects of Withaferin A combine with SHP2/MEK inhibition on KPC tumors *in vitro* and *in vivo*. (**a**) Increase in lipid peroxidation (C11-BODIPY fluorescence) in human and murine PDAC cell lines. Data shown are from a single biological sample, measured in one to two technical replicates. (**b**) Individual KPC mouse pancreatic volume changes over the course of the preclinical therapy trial, relative to baseline volume. (**c**) Quantification of MDA histoscores in KPC tumors treated with vehicle, the combination of SHP099 (75 mg/kg) and trametinib (1 mg/kg), or the combination of SHP099 (75 mg/kg) and trametinib (1 mg/kg) and withaferin A (4 mg/kg) administered together every other day starting at tumor detection. Animals marked with a square symbol were initially treated with SHP099 and trametinib after tumor detection and received additional withaferin A upon tumor relapse. Each symbol represents the histoscore of a single mouse, calculated as the mean of five to ten tumor regions analyzed per animal. (**d**) Body weights of mice over time under the indicated treatment conditions. Error bars indicate the standard error of the mean (SEM). (**e**) Frequencies of incidences of predefined study termination criteria stratified by treatment arms in the preclinical KPC trial.
